# CVD Grown Hybrid MoSe_2_–WSe_2_ Lateral/Vertical Heterostructures With Strong Interlayer Exciton Emission

**DOI:** 10.1002/adma.74172

**Published:** 2026-07-25

**Authors:** Md Tarik Hossain, Sai Shradha, Axel Printschler, Julian Picker, Luc F. Oswald, Julian Führer, Nicole Engel, Honey Jayeshkumar Shah, Christof Neumann, Daria I. Markina, Moritz Quincke, Johannes Biskupek, Kenji Watanabe, Takashi Taniguchi, Ute Kaiser, Bernhard Urbaszek, Andrey Turchanin

**Affiliations:** ^1^ Institute of Physical Chemistry Friedrich Schiller University Jena Jena Germany; ^2^ Institute of Condensed Matter Physics Technical University Darmstadt Darmstadt Germany; ^3^ Central Facility of Electron Microscopy, Electron Microscopy Group of Material Science Ulm University Ulm Germany; ^4^ Research Center for Electronic and Optical Materials National Institute for Materials Science Tsukuba Japan; ^5^ Research Center for Materials Nanoarchitectonics National Institute for Materials Science Tsukuba Japan; ^6^ Abbe Center of Photonics Jena Germany

**Keywords:** 2D materials, CVD, interlayer excitons, liquid precursors, MoSe_2_–WSe_2_ lateral and vertical heterostructures, transition metal dichalcogenide monolayers

## Abstract

Lateral heterostructures (LHSs) of 2D transition metal dichalcogenides (TMDs) offer a powerful platform to investigate photonic and electronic phenomena at atomically sharp interfaces. However, their controlled engineering, including tuning lateral domain size and integration into vertical van der Waals heterostructures with other 2D materials, remains challenging. Here, we present a facile route for the synthesis of two types of heterostructures (HSs), consisting of monolayers (MLs) of MoSe_2_ and WSe_2_—purely lateral (HS I) and hybrid lateral/vertical (HS II)—using liquid precursors of transition metal salts and chemical vapor deposition (CVD). Depending on the growth parameters, the heterostructure type and its lateral dimensions can be adjusted. We characterized properties of the HS I and HS II by complementary spectroscopic and microscopic techniques, including Raman and photoluminescence (PL) spectroscopy, optical and atomic force microscopy (AFM), and scanning and transmission electron microscopy (TEM). The PL measurements reveal strong interlayer exciton (IE) emission in the MoSe_2_/WSe_2_ region of HS II, which dominates the spectrum at 4 K and persists up to room temperature (RT). These results demonstrate high optical quality of the grown HSs, which in combination with the scalability of the developed approach, pave the way for fundamental studies and device applications based on these unique 2D quantum materials.

## Graphical abstract

A scalable bottom‐up CVD methodology, based on liquid transition metal salt precursors, is presented for the controlled growth of two high‐quality MoSe_2_–WSe_2_ heterostructures (HSs): purely lateral (HS I) and hybrid lateral/vertical (HS II), by simply adjusting precursor concentration. The heterobilayers in HS II exhibit strong interlayer excitonic emissions from 4 K to room temperature (RT).

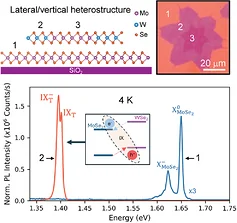

## 1 Introduction

Due to their atomic thickness and unique electronic and photonic properties, heterostructures (HSs) of monolayer (ML) transition metal dichalcogenides (TMDs), in both lateral and vertical configurations, hold significant promise for novel electronic, optoelectronic, and photonic devices as well as for studying new quantum phenomena [1, 2]. They enable investigations of new solid‐state phenomena like interlayer excitons (IEs) in vertical HSs [3] and charge‐transfer excitons in lateral HSs [4], electric transport through 1D *p–n* junctions [5], which are not accessible in individual TMD MLs. Based on the similarity of the crystal structures, 2D TMD can be stitched or stacked to form TMD_1_‐TMD_2_ lateral junction or vertical van der Waals TMD_1_/TMD_2_ HSs. Both HSs offer new opportunities for photonics, hosting various excitonic phenomena and enabling their device integration (see e.g., [3, 6, 7, 8]). For instance, lateral HSs (LHs) demonstrate unidirectional in‐plane exciton transport [9], which facilitates the development of excitonic devices. The spatial modulation of electronic properties in LHs enables the formation of atomically thin in‐plane *p–n* junctions for the engineering of transistors, diodes, photodetectors, photovoltaic devices, electroluminescent devices, and other quantum devices [5, 10, 11, 12, 13]. On the other hand, vertical HSs demonstrate the formation of IEs, where electrons and holes are separated between the two adjacent TMD MLs (see e.g., [3, 14, 15, 16, 17]). As an example, MoSe_2_/WSe_2_ vertical HS form the type‐II band alignment, resulting in a rapid interlayer charge transfer between MoSe_2_ and WSe_2_ and in the formation of IEs with long lifetimes [3, 18, 19], which facilitates the development of fields such as quantum encryption and valleytronics [20, 21, 22, 23]. Furthermore, Moiré‐trapped IEs, owing to their discrete emission, may be used as single photon emitters [23] and for lasing via coupling to nanocavities [24].

For fundamental studies and applications, engineering of TMD HSs by bottom‐up approaches is particularly important as it enables scalability and integration into semiconductor and optical technologies. Typically, either one‐step [7, 25, 26, 27, 28, 29, 30, 31] or two/multi‐step [13, 32, 33, 34] chemical vapor deposition (CVD) growth methods using powders of transition metal oxides (e.g., MoO_3_ and WO_3_) and chalcogens (S and Se) or a mixture of transition metal dichalcogenide (e.g., WSe_2_ and MoSe_2_) as precursors are utilized to this end [2, 35]. In a more conventional two‐step growth method, MLs of two different TMDs are grown sequentially employing separate growth reactors [32]. Such a method poses challenges, as the exposure of the first TMD to the ambient conditions before the growth of the second one can compromise the quality of the atomic interfaces. Alternatively, a one‐step CVD enables the growth within the same reactor without exposing the sample to ambient conditions. However, the use of powder metal oxides limits the ability to tune the HS sizes due to difficulties in controlling their vapor pressures and fluxes of the precursors during the growth. On the other hand, water‐soluble sodium molybdate (Na_2_MoO_4_) and sodium tungstate (Na_2_WO_4_) salts provide flexibility in tuning the transition‐metal ratios in the precursors by their appropriate mixing and therefore possess great promise to overcome this limitation. To the best of our knowledge, up to date, only a few studies have reported CVD growth of HSs using liquid precursors [36, 37, 38, 39]. For instance, metal–organic liquid precursors have been employed to grow TMD_1_‐TMD_2_ lateral HSs [36], while ammonium‐ and sodium‐based salts have been used for the growth of MoS_2_–WS_2_ lateral [39] and vertical HSs to investigate their electrical transport properties [37]. Similarly, sodium‐based salts have enabled the synthesis of MoSe_2_–WSe_2_ lateral HSs for photodetector applications [38]. Nevertheless, the realization of both vertical and lateral HS with clean and atomically sharp interfaces for studying excitonic phenomena remains a challenge and in this respect bottom‐up approaches hold a significantly higher potential in comparison to their top‐down assembly from individual MLs, where contamination is intrinsically unavoidable.

Here, we demonstrate a tunable, highly reproducible, and scalable one‐step CVD approach, utilizing liquid precursors of transition metals, to grow purely lateral (HS I) as well as hybrid lateral/vertical (HS II) HSs of MoSe_2_ and WSe_2_ MLs, Figure 1. To obtain the desired HSs, aqueous solutions of Na_2_MoO_4_ and Na_2_WO_4_ salts with different concentrations were spin‐coated onto 300 nm SiO_2_/Si growth substrates, followed by a CVD conversion step at elevated temperatures in the presence of Se vapor. The formation of HS I and HS II, their sizes, and ratios of MoSe_2_ and WSe_2_ MLs’ areas can be tuned by the concentration of the salts and by the growth temperature. We employed complementary microscopic and spectroscopic techniques, including optical microscopy (OM), atomic force microscopy (AFM), Raman spectroscopy, photoluminescence (PL) spectroscopy, and transmission electron microscopy (TEM), including high‐resolution (HR) and dark field scanning (STEM) together with energy dispersive x‐ray spectroscopy (EDX) to study their morphological, structural, chemical, and optical properties. By PL measurements, we reveal over a broad temperature range (from 4 to 300 K) a strong IE emission in the WSe_2_/MoSe_2_ region of HS II. The room temperature (RT) IE formation is remarkable and demonstrates an efficient carrier transfer between the CVD‐grown WSe_2_ and MoSe_2_ MLs. It is also striking that the IE at *T* = 4 K dominates the PL spectrum, which has only been observed in the HSs obtained by mechanical stacking of individual TMD MLs exfoliated from bulk crystals [40] or grown via the CVD method from solid precursors [41, 42].

**FIGURE 1:**
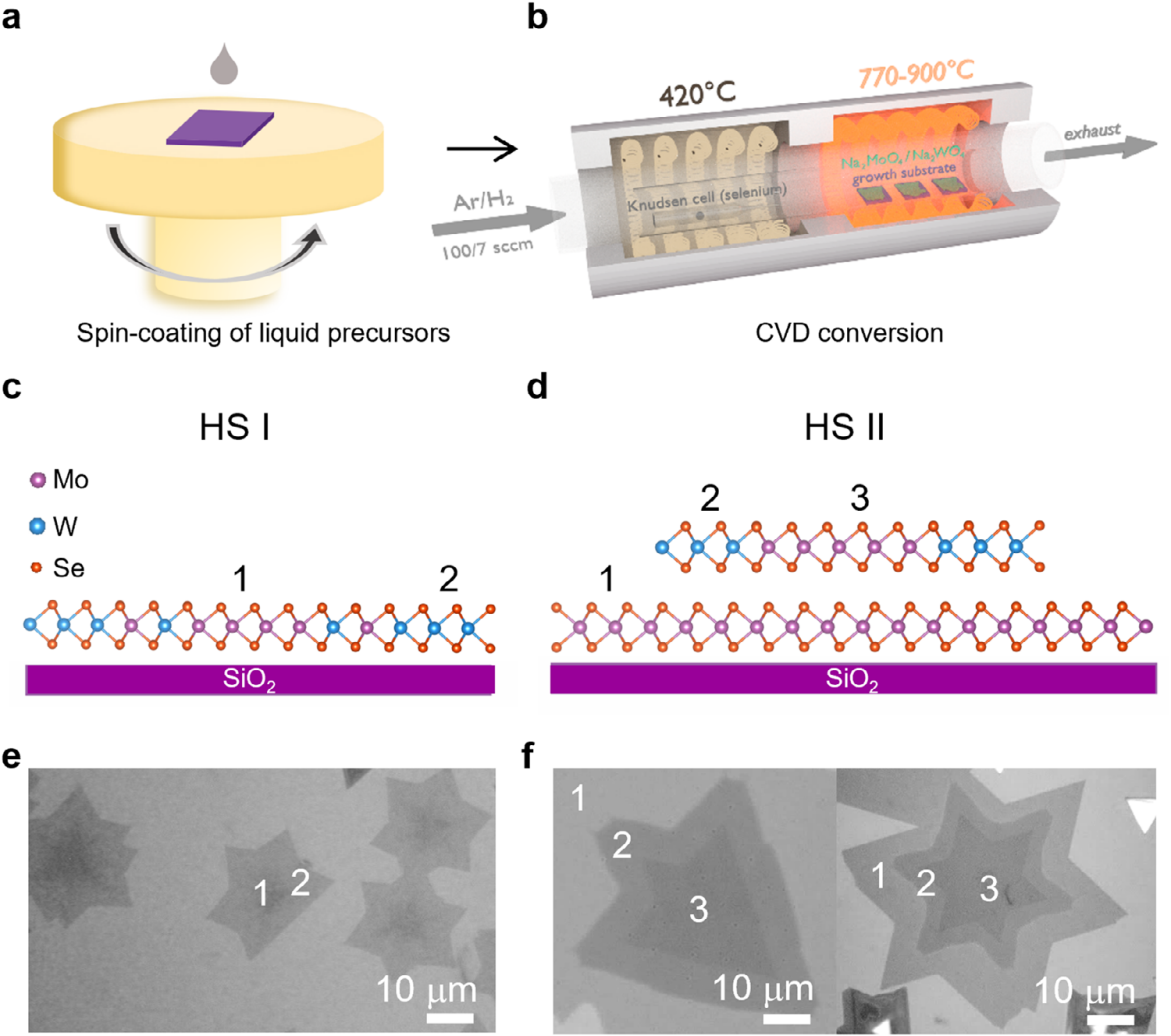
Growth steps and structural configurations of MoSe_2_–WSe_2_ HSs. (a,b) Schematics illustrating the growth process for MoSe_2_ and WSe_2_ HSs, which includes the preparation of precursor substrate and subsequent CVD conversion. (c,d) Schematics of two HS configurations: HS I, where MoSe_2_ and WSe_2_ are stitched laterally, and HS II, where a MoSe_2_ bottom layer is vertically stacked with a MoSe_2_–WSe_2_ lateral heterostructure (LH) grown on top. (e,f) The representative OM images of HS I and HS II, respectively.

## 2 Results and Discussion

### 2.1 CVD Growth of HS I and HS II Using Liquid Precursors

We begin with a general description of the preparation method of HS I and HS II illustrated in Figure 1a,b. To this end, the liquid Na_2_MoO_4_ and Na_2_WO_4_ precursors, which served as the molybdenum (Mo) and tungsten (W) sources, respectively, were deposited onto a 300 nm SiO_2_/Si growth substrate by spin‐coating and subsequently converted into the HSs in a CVD reactor where solid selenium powder (Se) was used as chalcogen precursor. By adjusting the Mo:W precursors' molar ratios (1:3 or 3:1; Table S1), two distinct configurations were obtained. HS I consists of a MoSe_2_ core laterally surrounded by WSe_2_, forming hexagonal, star‐like, or triangular crystals (Figure 1c,e and Figure S1). HS II exhibits a vertical bilayer structure with a MoSe_2_ base and a lateral MoSe_2_–WSe_2_ top layer with triangle or hexagonal edges (Figure 1d,f, and Figure S2). For growth, a modified CVD reactor equipped with quartz Knudsen‐type effusion cells was employed, thereby enabling precise control of the selenium vapor flux [43]. The reactor furnace was heated to growth temperatures of 770°C, 820°C, or 900°C at a rate of 15°C min^−1^ (Figure S3). Given the lower melting point [44] and the higher partial pressure of Mo species [5], MoSe_2_ nucleated first, followed by epitaxial WSe_2_ growth from the MoSe_2_ edges. At a higher Mo concentration (3:1), a bilayer MoSe_2_ island formed, which likely acted as a nucleation site for WSe_2_ growth and thereby promoted the growth of vertical HS II. In contrast, a lower Mo concentration (1:3) favored the growth of ML MoSe_2_ and lateral WSe_2_ stitching to form HS I. Individual MoSe_2_ growth at high and low concentrations of Na_2_MoO_4_, representing the 3 and 1 values in the precursor ratios, confirms this mechanism, yielding mainly MoSe_2_ with bilayer island or ML MoSe_2_, respectively (Figure S4). A heating rate of 15°C min^−1^ was essential for pure‐phase of MoSe_2_–WSe_2_ LHs formation, as faster heating rates (40°C min^−1^) led to mixed‐phase LHs (Figure S5), which is consistent with the fast kinetics of TMD MLs at our experimental conditions [44, 45]. Although the growth temperature did not affect preferential growth of HS I or HS II, at higher temperatures, the lateral sizes of HS I are increased (Figures S6 and S7). In addition, increasing the W precursor concentration expanded WSe_2_ domains by approximately two‐ to three‐fold (*Figure* *S7*), with the entire lateral sizes of HS I ranging from ∼10 to 200 µm (Figures S7 and S8) and of top LH of HS II ranging from ∼20 to 90 µm (Figures S6a, S7b and S9b). Thus, using liquid precursors, both HS I and HS II can be grown uniformly across the substrate with surface coverage of ∼50%–90% (Figures S8a and S9a); further technical details of the HS growth are presented in the Experimental Section.

### 2.2 Spectroscopic and Microscopic Characterization of HS I and HS II Down to the Nanoscale

To study the properties of the formed HS I and HS II, we applied several complementary microscopic and spectroscopic techniques at RT. We first conducted Raman spectroscopy on the inner and outer regions of lateral HS I to identify the MLs and their structural quality (Figure 2a). The inset of Figure 2a shows an OM image indicating the measurement locations. The characteristic Raman peaks at 240 cm^−1^ (A′_1_ mode) and 286 cm^−1^ (E′ mode) confirm the presence of MoSe_2_ in the inner region. Peaks at 249 cm^−^^1^ (E′/A′_1_ modes) and 260 cm^−^^1^ (2LA mode) correspond to the WSe_2_ in the outer region. The narrow full‐width at half maximum (FWHM) values of the A′_1_ mode of MoSe_2_ (1.7 cm^−^^1^) and the E′/A′_1_ modes of WSe_2_ (3.7 cm^−^^1^) indicates high‐crystalline quality of the HSs grown from liquid precursors, which is comparable to those grown from solid precursors of transition metals [5]. To assess the uniformity of these lateral HSs, Raman intensity maps were recorded for the A′_1_ mode of MoSe_2_ and the E′/A′_1_ mode of WSe_2_ across the entire MoSe_2_–WSe_2_ HS I (Figure 2b,c). The A′_1_ peak intensity map of MoSe_2_ (Figure 2b) reveals a uniform signal within the inner region of the HS, while the E′/A′_1_ peak intensity map of WSe_2_ (Figure 2c) shows localization in the outer region only. These results confirm the well‐defined formation of lateral HS I. Further, AFM studies show (Figure S10) a thickness of ∼0.8 nm, indicating a ML structure.

**FIGURE 2:**
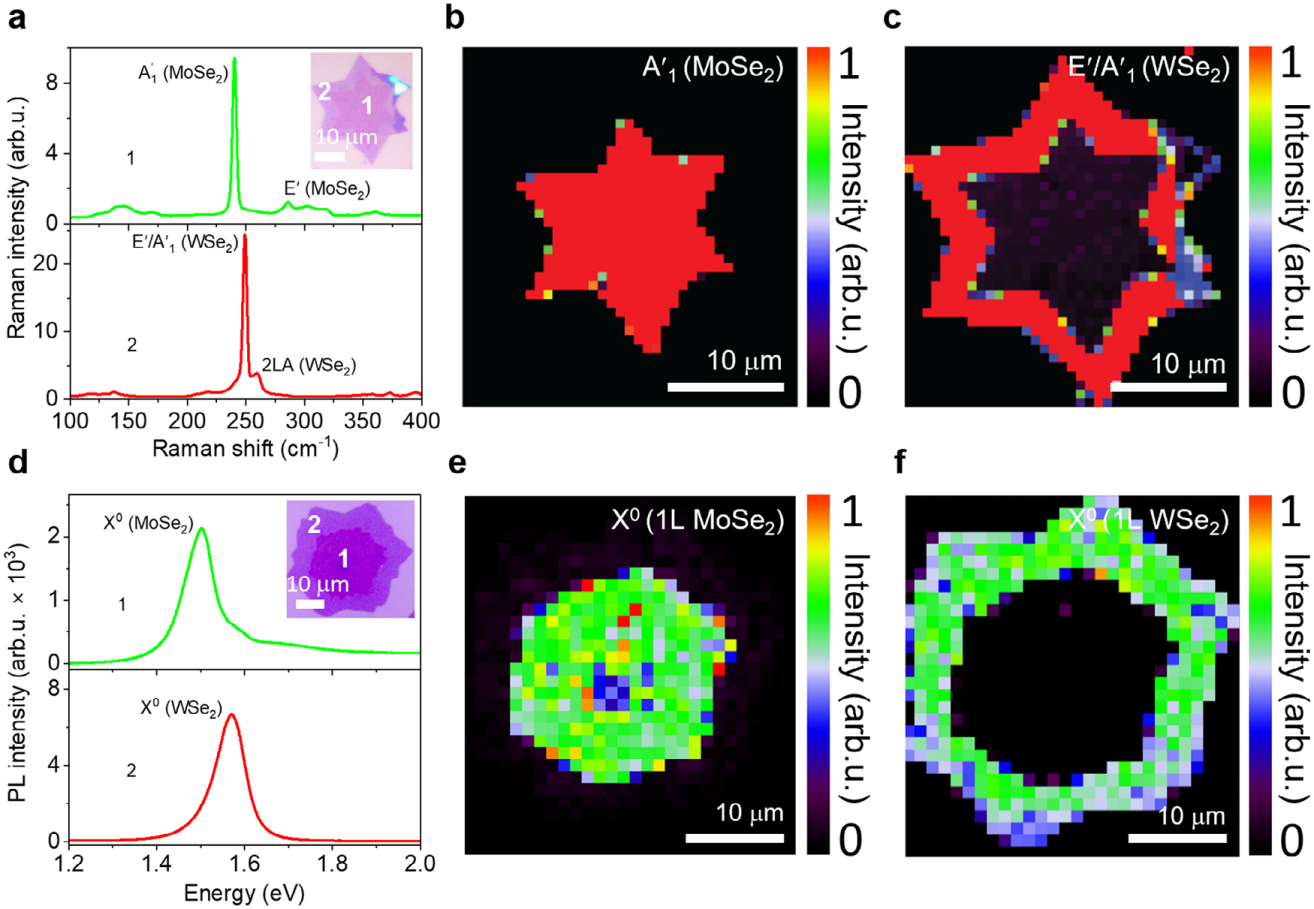
Raman spectroscopy and PL characterization of HS I at RT. (a) Raman spectra acquired from region 1 (MoSe_2_) and region 2 (WSe_2_), with the inset displaying the OM image indicating the measurement positions. (b) Raman intensity map of A′_1_ mode (MoSe_2_) recorded over the area shown in the inset of (a), showing uniform crystallinity in the inner region. (c) Raman intensity map of the E′/A′_1_ mode (WSe_2_) showing uniform crystallinity in the outer region. (d) PL spectra of inner MoSe_2_ (bottom panel) and outer WSe_2_ (top panel). The inset shows the OM image indicating the corresponding measurement positions. (e,f) PL intensity map of MoSe_2_ and WSe_2_, collected over the area shown in the inset of Figure (d).

To evaluate the optical properties of the lateral HS I, we performed RT PL measurements using a 532 nm laser. The PL spectra (Figure 2d) show a ML MoSe_2_ emission peak at 1.50 eV (neutral exciton, X^0^) and a ML WSe_2_ emission peak at 1.57 eV (X^0^). Both emission energies are significantly lower than the typically reported values of 1.57 eV (MoSe_2_) and 1.65 eV (WSe_2_) [3], suggesting that the HSs are under tensile strain, which was likely introduced during the CVD process due to the different substrate/TMD thermal expansion coefficients [46]. Despite this strain effect, the strong PL intensity indicates that HS I remains a direct bandgap and of high optical quality. The PL intensity of the WSe_2_ region is approximately 3.5 times stronger than that of MoSe_2_, consistent with a previous report on lateral MoSe_2_–WSe_2_ HS grown from solid precursors (mixtures of MoO_3_ and WO_3_ powders) [47]. Figure 2e,f presents spatial PL maps of lateral MoSe_2_–WSe_2_ HS I. The PL mapping at 1.50 eV (Figure 2e) clearly highlights the inner MoSe_2_ region, while the 1.57 eV map (Figure 2f) identifies the outer WSe_2_ region.

To elucidate the nanoscale interface structure of HS I, we performed high‐angle annular dark‐field (HAADF) STEM, where images are sensitive to atomic‐number (Z) contrast, in combination with energy‐dispersive x‐ray spectroscopy (EDX) elemental mapping (Figure 3a–c and Figure S12). As expected, the EDX elemental maps (Figure 3b and Figure S12) show a uniform distribution of Se across the entire HS I, while Mo is localized in the centre and W in the outer regions. The contrast differences in the overview HAADF‐STEM image (Figure 3a, darker contrast for lighter Mo atoms and brighter contrast for heavier W atoms) clearly support the EDX results and reveal additional details—an intermediate Mo─W─Se region of approximately 120 nm in width between MoSe_2_ and WSe_2_, where both transition metals are present, indicating alloy formation. High‐resolution HAADF‐STEM further shows that the interfaces between MoSe_2_/Mo─W─Se (Figure 3c) and Mo─W─Se/WSe_2_ (Figure S13) are confined to only **≈** 5 nm. The sharp transition between the alloy and pure materials, together with the wider alloyed region, is possibly driven by nucleation kinetics combined with substrate‐induced effects in our one‐step CVD growth. Selected‐area electron diffraction (SAED) patterns acquired from these three distinct regions (Figure 3d–f) show sharp six‐fold symmetry, and with nearly identical {10–10} d‐spacings, demonstrating a single crystalline in‐plane heterojunction in HS I.

**FIGURE 3:**
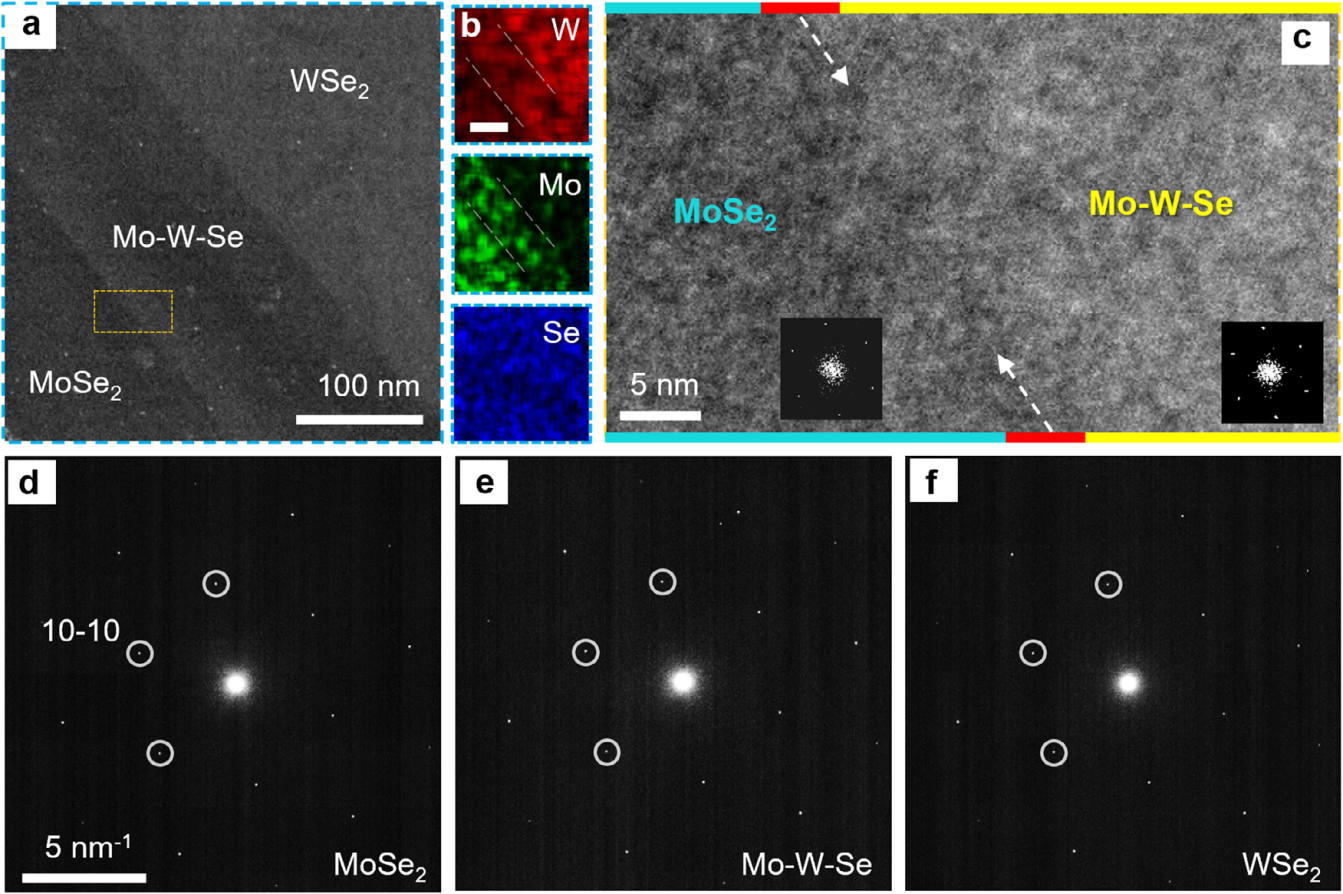
Nanoscale characterization of HS I interface. (a) 200 kV HAADF‐STEM image of the HS interface, revealing an ∼120 nm Mo─W─Se region bridging the MoSe_2_ and WSe_2_ domains. (b) Elemental maps obtained from (a) of W, Mo, and Se highlight the compositional transition across the intermixed, alloyed region. (c) High‐magnification 200 kV HAADF‐STEM image of the MoSe_2_ and Mo─W─Se boundary shows a narrow ∼5 nm interface (red lines), with MoSe_2_ and Mo─W─Se regions indicated by cyan and yellow lines, respectively. A white dashed arrow is included as a guide to the eye. The inset displays the corresponding fast Fourier transforms (FFTs) obtained from the respective regions. (d–f) SAED patterns obtained at 200 kV with the smallest SAED aperture of 10 µm, resulting in an about 100 nm wide area from the MoSe_2_, Mo─W─Se, and WSe_2_ regions, respectively. The {10–10} plane d‐spacings are 0.2818 nm (MoSe_2_), 0.2819 nm (Mo─W─Se), 0.2821 nm (WSe_2_) (see the WSe_2_ HAADF image in Figure S13).

Next, we characterize the properties of HS II samples. As introduced in Section 2.1, HS II is a bilayer configuration with a bottom MoSe_2_ ML and a lateral MoSe_2_–WSe_2_ top layer. Raman spectroscopy (see Figure 4a) at three different regions (marked with 1, 2, and 3 in the inset of Figure 4a, also see Figure 1d) identifies the layers of such HS II. In region 1 (bottom layer), the Raman modes at 240 (A′_1_) cm^−1^ and 286 (E′) cm^−1^ confirm the MoSe_2_ ML, consistent with the peak positions observed in ML MoSe_2_ of HS I. In region 2 (heterobilayer region), the Raman modes at 241 (A′_1_ of MoSe_2_) cm^−1^, 249 (E′/A′_1_ of WSe_2_) cm^−1^, and 259 (2LA of WSe_2_) cm^−1^ confirm the vertical HS of MoSe_2_/WSe_2_. In region 3 (homobilayer region), the characteristic Raman modes at 241 (A′_1_) cm^−1^ and 286 (E′) cm^−1^ confirm the MoSe_2_. Noticeably, the A′_1_ mode of MoSe_2_ (compared between region 1 and region 3) shows a discernible blueshift, as expected in bilayer MoSe_2_ because of weak van der Waals interactions [48]. Raman intensity maps of the A′_1_ mode of MoSe_2_ and E′/A′_1_ mode of the WSe_2_ on the entire lateral/vertical MoSe_2_–WSe_2_ HS II are depicted in Figure 4b and Figure 4c, respectively. The intensity of the A′_1_ peak of MoSe_2_ (Figure 4b) is primarily detected in region 1 and region 3 of the HS, with a lower contrast in region 2, as expected. The intensity map of the E′/A′_1_ peak of WSe_2_ (Figure 4c) shows that WSe_2_ is only present in region 2, validating the vertical MoSe_2_/WSe_2_ HS formation. The AFM height profile across the bottom MoSe_2_ ML and top lateral MoSe_2_–WSe_2_ HS (Figure S14) shows an estimated height of ∼ 0.8 nm, further confirming the ML of lateral MoSe_2_–WSe_2_ on top of the MoSe_2_ ML.

**FIGURE 4:**
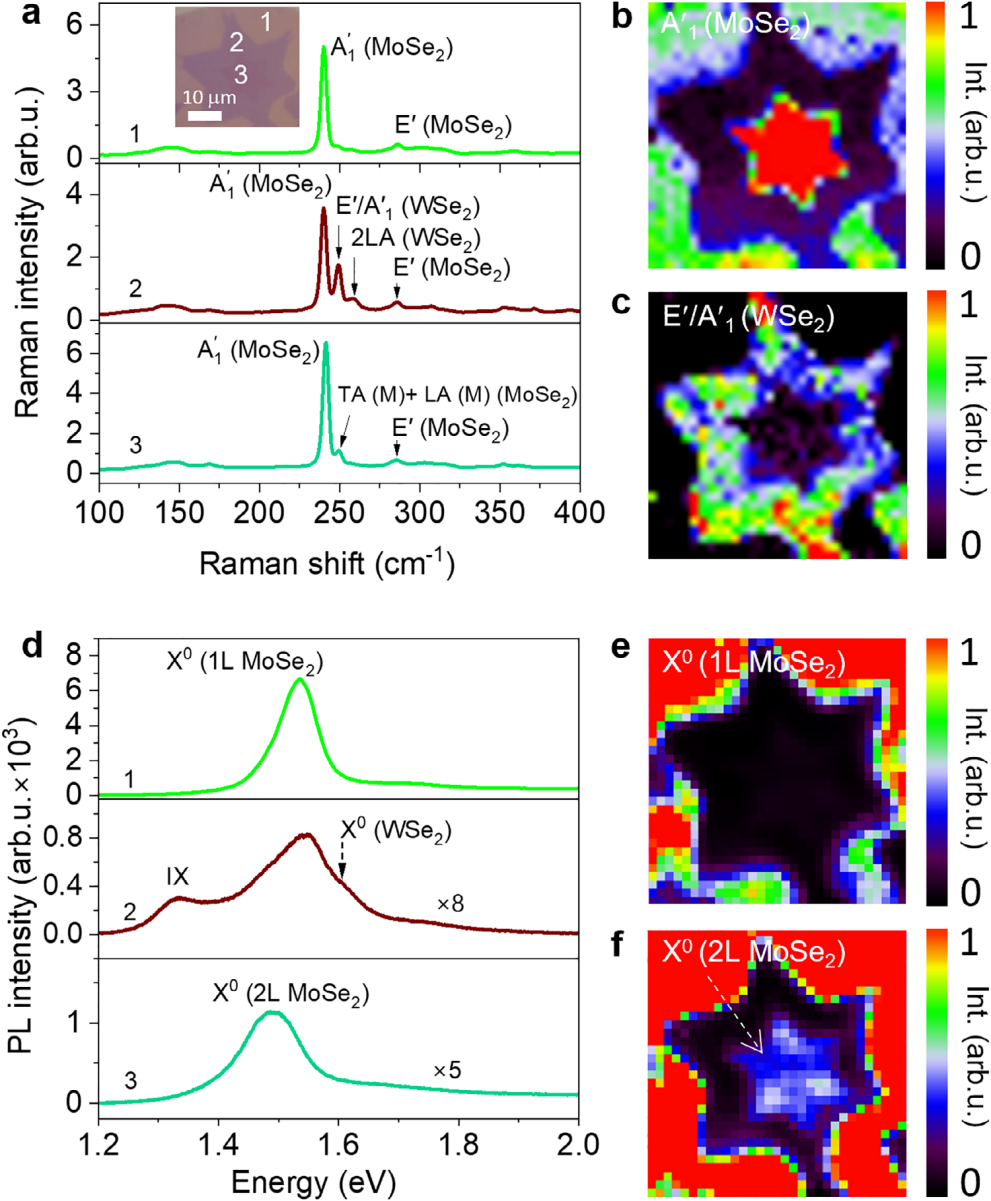
Raman spectroscopy and PL characterization of HS II at RT. (a) Raman point spectra at regions 1 (1L MoSe_2_), 2 (vertical MoSe_2_–WSe_2_ bilayer), and 3 (2L MoSe_2_) as shown in the OM image of hybrid lateral‐vertical MoSe_2_–WSe_2_ HS II in the inset. (b) and (c) show the Raman intensity spatial maps of A′_1_ of MoSe_2_ and E′/A′_1_ of WSe_2_, respectively. (d) PL spectra at the three phases, which were collected from three different regions of the inset of Figure (a). (e) and (f) show the corresponding PL intensity map of 1L MoSe_2_ (1.54 eV) and 2L MoSe_2_ (1.49 eV) in the PL spectra in (d).

Next, we analyse the PL spectra of HS II. As seen from Figure 4d, the PL intensity at 1.54 eV (X^0^) from bottom ML MoSe_2_ (region 1) in comparison to that at 1.49 eV (X^0^) from bilayer MoSe_2_ (region 3) is ∼ thirty times higher as expected for a direct and nondirect band gap semiconductors [49], respectively. From the value of the X^0^ emission energy for the ML MoSe_2_, some substrate‐induced tensile strain is expected [50], which is lifted if HS II is encapsulated in hBN (see Section 2.3). Most prominent is that in region 2, the PL spectrum also reveals the IE emission at ∼1.33 eV (IX), see middle spectrum in Figure 4d and Figure S15, due to type‐II band alignment between MoSe_2_ and WSe_2_ [3]. Additionally, Figure 4e,f represents the PL mapping of the HS II at 1.54 and 1.49 eV, which clearly reflect regions 1, 2, and 3 of the HS II HS.

To characterize the nanoscale structure of HS II, we performed HAADF‐STEM imaging combined with EDX analysis (Figure 5 and Figure S16). The EDX data from region 2 reveal nearly equal atomic fractions of Mo and W, consistent with a vertical heterobilayer of MoSe_2_ and WSe_2_, whereas region 1 and region 3 are dominated by the Mo signal. Examination of the MoSe_2_–WSe_2_ lateral interface shows an interface width of only **≈** 5 nm (Figure 5a–c). We attribute this sharp interface to the epitaxial growth of the top lateral HS on the bottom MoSe_2_ ML. This interpretation is supported by the continuous atomic rows with no visible Moiré pattern in the HRTEM image (Figure 5d and Figure S17a) and by the SAED patterns showing a single set of sharp circular Bragg spots (Figure 5e and Figure S18), both of which are direct evidence of epitaxial alignment. In contrast, nonepitaxial growth of the second layer, or a twisted bi‐layer, would typically produce Moiré‐fringes in HRTEM images and split diffraction spots in the SAED.

**FIGURE 5:**
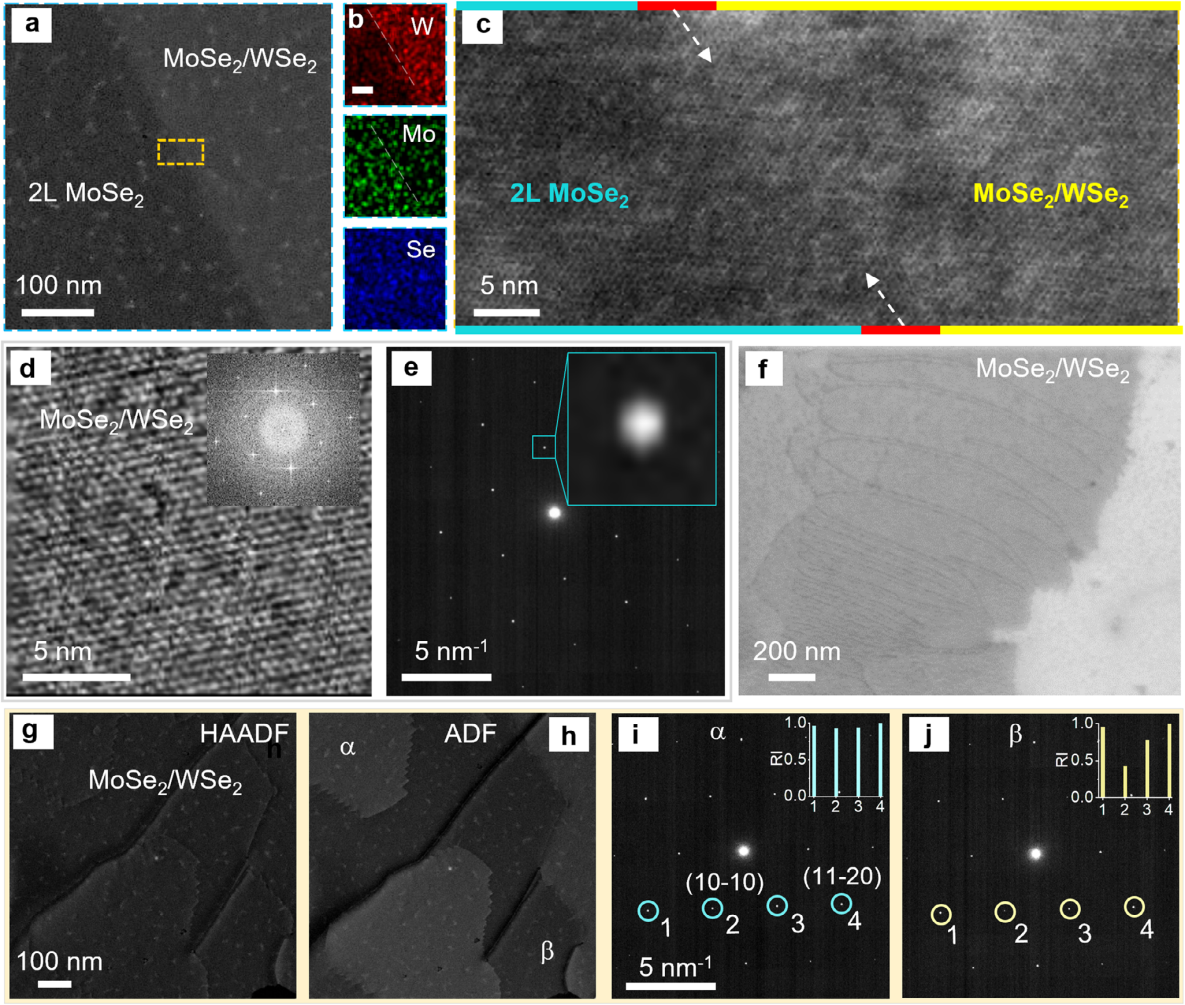
Nanoscale characterization of HS II structure. (a) 200 kV HAADF‐STEM image of the interface of the LH region. (b) EDX elemental maps of W, Mo, and Se of the image in (a). (c) 200 kV HR HAADF‐STEM image showing the ∼5 nm interface between the LH MoSe_2_–WSe_2_ regions. (d) 200 kV HRTEM image of the vertical MoSe_2_/WSe_2_ HS region. The inset represents the FFT. (e) The SAED pattern acquired at 200 kV exhibits six‐fold symmetry with single circular diffraction spots, which indicates epitaxial growth of the top LH. The inset shows a magnified view of a single diffraction spot. (f) SEM micrograph of the MoSe_2_/WSe_2_ region of HS II on 300 nm SiO_2_/Si substrates reveals the reconstruction patterns. (g,h) Comparison of 200 kV HAADF‐ and ADF‐STEM images of the MoSe_2_/WSe_2_ region, where domains of brighter and darker contrast are clearly resolved in the ADF image, labeled as *α* and *β*, respectively. (i,j) Corresponding 200 kV SAED patterns at domains *α* and *β* with insets showing intensities of first (positions 2,3)‐ and second (positions 1,4)‐order diffraction spots, respectively, indicating the H (*α*) and R (*β*)‐ like stacking domains.

A small lattice mismatch between MoSe_2_ and WSe_2_ facilitates reconstruction phenomena in the vertical stacks of the constituent MLs, giving rise to rich excitonic features [51]. We employed scanning electron microscopy (SEM) to investigate the mesoscopic reconstruction of MoSe_2_/WSe_2_ heterobilayer region in HS II on a SiO_2_/Si substrate. The SEM image (Figure 5f) reveals a pattern of one‐dimensional (1D) stripes, characteristic of such reconstruction [51]. To further determine the stacking order, we combined HAADF, annular dark field (ADF) imaging, and localized SAED measurements (Figure 5g–j). Typically, HAADF contrast is dominated by Z‐contrast [52], whereas ADF contrast [53], and the relative diffraction spots intensities in SAED provide insight into stacking order [54, 55]. In both HAADF and ADF images (see Figure 5g,h), we observe 1D stripes consistent with the reconstructed pattern seen in SEM (Figure 5f). In addition, the ADF image reveals brighter and darker domains (Figure 5h) that are not clearly resolved in the HAADF image (Figure 5g), indicating that the top WSe_2_ ML forms domains of different stacking orders on the bottom MoSe_2_ ML. We label these domains *α* (brighter) and *β* (darker) and acquired the SAED patterns from each domain. The SAED pattern from domain *α* (Figure 5i) shows comparable intensities of the first‐ and second‐order diffraction spots (inset of Figure 5i), consistent with AB (H‐like) stacking [54, 55]. In contrast, the SAED pattern from domain *β* (Figure 5j) exhibits markedly weaker first‐order diffraction intensities relative to the second‐order spots (inset of Figure 5j), indicating AA (R‐like) stacking [54, 55]. HRTEM imaging (Figure S19) further supports the coexistence of AB (H‐like) and AA (R‐like) stacking, demonstrating that the heterobilayer in HS II contains a hybrid of both stacking domains. Thus, the heterobilayer exhibits mesoscopic reconstruction and mixed stacking domains.

### 2.3 Strong IE Emission at MoSe_2_/WSe_2_ Heterobilayer in HS II Across 4–300 K Range

Previous work on CVD‐grown samples has shown the benefit of hBN encapsulation [56, 57, 58]. As the samples are lifted off their growth substrates, the layers are essentially unstrained [59]. This is evidenced by the RT PL spectral map of the hBN‐encapsulated HS II (see Figures S20 and S21a–e), which shows uniform, strain‐free emission energies across the HS. To investigate the HS optically, we have performed PL measurements. In Figure 6a, we show the PL emission from the MoSe_2_/WSe_2_ heterobilayer region at T = 4 K. Here, distinct emission corresponding to interlayer excitonic species is observed. These peaks labeled ${\mathrm{IX}}_{T}^{-}$ (1.395 eV) and *IX_T_* (1.402 eV) are attributed to the charged and neutral spin‐triplet IEs [40, 60]. In this region of the sample, no emission from intralayer species is discernible as seen in Figure 6a. This dominance of the interlayer emission indicates very efficient transfer of electrons from WSe_2_ and holes from MoSe_2,_ leading to the formation of IEs (as represented in the inset of Figure 6a). Such a strong signature of the IE is a promising characteristic of liquid precursor‐grown heterobilayers. Figure 6b shows the PL emission from 1L‐MoSe_2_ region at T = 4 K where the contributions are from trion ($X_{MoSe_{2}}^{-}$) and neutral ($X_{MoSe_{2}}^{0}$) excitons [61, 62]. As a control, we compared the PL properties of our CVD‐grown MoSe_2_/WSe_2_ HS in HS II with those of an exfoliated MoSe_2_/WSe_2_ HS (Figure S22). The CVD‐grown sample exhibits significantly narrower IE peaks, with FWHM values reduced by ∼ 37% for the ${\mathrm{IX}}_{T}^{-}$ (${\mathrm{IX}}_{T\;}^{-}\left(CVD\right)$= 6.8 ± 0.13 meV vs. ${\mathrm{IX}}_{T\;}^{-}\left(Exf.\right)$= 10.78 ± 0.57 meV) and ∼ 67% for the ${IX}_{T}$ (${IX}_{T\;}\left(CVD\right)$ = 4.27 ± 0.06 meV vs. ${IX}_{T\;}\left(Exf.\right)$ = 13.03 ± 0.21 meV), compared to the exfoliated sample. These results highlight the superior optical quality and atomically cleaner vertical interface of the CVD‐grown HS. In addition, ∼ 54% linewidth reduction in the 1L‐MoSe_2_ intralayer exciton (from $X_{MoSe_{2}}^{0}\left(Exf.\right)$ = 5.67 ± 0.12 meV to $X_{MoSe_{2}}^{0}\left(CVD\right)$= 2.6 ± 0.02 meV) indicates excellent optical quality even in the ML MoSe_2_ region 1 of HS II.

**FIGURE 6:**
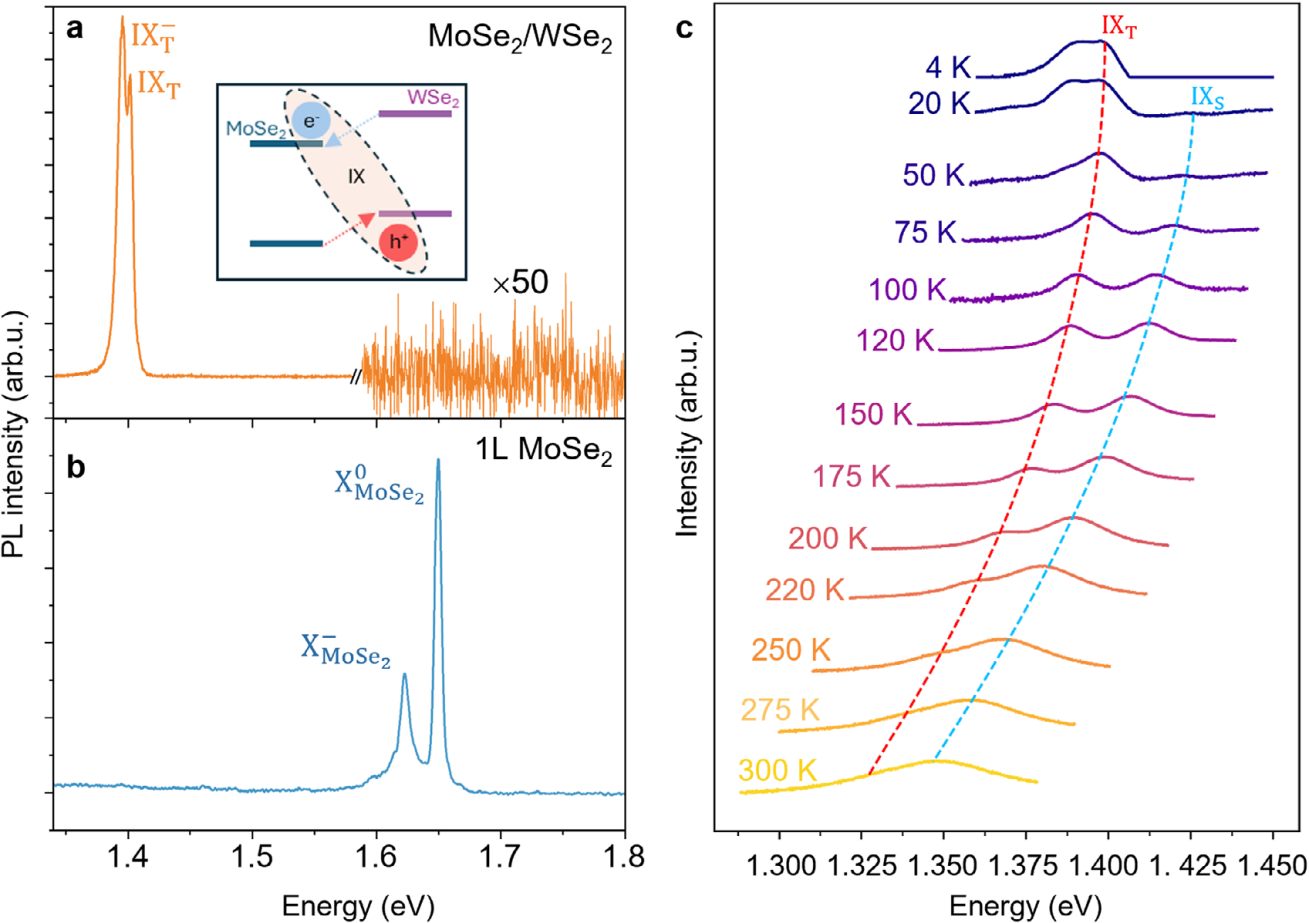
PL spectroscopy of IEs in hBN encapsulated HS II: Emission at T = 4 K from (a) the MoSe_2_/WSe_2_ heterobilayer showing the neutral (*IX_T_*) and charged ($IX_{T}^{-}$) spin‐triplet IEs and (b) the 1L‐MoSe_2_ with contributions from the trion ($X_{MoSe_{2}}^{-}$) and neutral ($X_{MoSe_{2}}^{0}$) exciton species. (c) Temperature‐dependent PL from the heterobilayer region with Varshni fits. *IX_T_* shows a discernible emission from 4 to 300 K. $IX_{T}^{-}$ vanishes above 20 K. Above 20 K, additional peak appears in the interlayer emission spectral region owing to the spin‐singlet state *IX_S_*. At temperatures below 75 K, the PL emission is dominated by *IX_T_*, however, at higher temperatures, the singlet state, *IX_S_* dominates the emission.

Most studies on IEs focus on spectroscopy at cryogenic temperatures. But in Figure 4d, a spectral feature at the IE energy seems to be discernible at RT. This motivated our temperature‐dependent measurements to follow the evolution of the PL emission from *T* = 4–300 K. The results are summarized in Figure 6c. It can be seen that we are indeed able to trace the IE emission at all temperatures between 4 and 300 K. For temperatures between 4 and 75 K, the contribution to the PL is mainly from emissions ${\mathrm{IX}}_{T}^{-}$ and IX_T_. At temperatures above 20 K, a higher energy contribution corresponding to the spin‐singlet interlayer excition (IX_s_) is detected [40]. From around 50 K onward, the emission of ${\mathrm{IX}}_{T}^{-}$ is no longer detectable, possibly indicating weak localization [63]. Following the interlayer emission through increasing temperatures, it is observed that the singlet state IX_s_ gains intensity and becomes the dominant peak at 300 K. This has certain parallels to the temperature evolution of the intralayer exciton PL in ML WSe_2_, where the spin‐allowed bright exciton state becomes more intense with temperature [64]. The singlet state IX_s_ emission also corresponds to the RT transition energy observed in Figure 4d. Globally, the IE emission dominates the PL in region 2 of HS II for all temperatures investigated. Furthermore, as can be seen in Figure 6c, the individual emission energies are fitted with Varshni functions [65, 66]. The fits are indicated as dashed lines in the plot. All the peak shifts are in good agreement with the Varshni function. Thus, showing that we can track the IE emission over the entire temperature range. This excludes defects from being the origin of the low temperature PL. The temperature evolution of the IE emission energy depends on the valence band shift in WSe_2_ and the conduction band shift in MoSe_2_. The Varshni parameters for intralayer MoSe_2_, WSe_2,_ and IEs are compared in Table S2 (Supporting Information). The PL characteristics reported in this section correspond to 2H stacking for the sample investigated, as the attribution of exciton lines in excellent agreement with the literature [40, 60].

## 3 Conclusions

In summary, we demonstrate a scalable bottom‐up CVD methodology, based on liquid transition metal salts precursors, for the controlled growth of two high‐quality MoSe_2_–WSe_2_ HSs: HS I and HS II. By simply adjusting precursor concentration, we selectively direct the preferential growth of HS I and HS II structures. We found that the vertical MoSe_2_/WSe_2_ heterobilayers in HS II exhibit strong interlayer excitonic emission that remains visible at RT and becomes the sole contributor at 4 K—a promising feature that indicates exceptionally clean and atomically sharp interfaces of these samples. We envision that our simple CVD growth process of HS I and HS II from liquid precursors will enable the rapid synthesis of these complex HSs for excitonic devices, which can readily offer a scalable alternative to manually assembled HSs and can be integrated into nanoelectronic and photonic devices.

## 4 Experimental

### 4.1 Sample Preparation

#### 4.1.1 Materials

Sodium molybdate (Na_2_MoO_4_ powder, 98.0%, Sigma–Aldrich), sodium tungsten oxide dihydrate (Na_2_WO_4_, 2H_2_O powder, 99.0%, Alfa Aesar), and selenium granules (99.9%, Sigma–Aldrich) were used without further purification.

#### 4.1.2 Aqueous Sodium Molybdate and Sodium Tungstenate Solution Preparation

For the molybdenum (Mo) and Tungsten (W) source, the aqueous solution of sodium molybdate and sodium tungstenate was prepared in ultrapure water (0.056 µS/cm, MembraPure Aquinity2 E35).

#### 4.1.3 Precursor Substrates Preparation

Approximately one and a half centimeters by one and a half centimeters Si substrate with a 300 nm thick SiO_2_ layer (Sil'tronix, root mean square (rms) roughness < 0.2 nm) was used as growth substrate. To improve the substrate's hydrophilicity and increase the wettability of Mo/W precursor during spin coating, the substrates were exposed to O_2_ (10 sccm) plasma for 280 s (Diener Zepto). Further, the aqueous solutions of sodium molybdate and sodium tungstenate, primarily in 1:3, and 3:1 ratios, were spin‐coated at 2000 rpm on plasma‐cleaned SiO_2_/Si substrate, followed by heating at 70°C for 5 min. Additional precursor ratios were processed under identical conditions to obtain controlled growth statistics (data presented in Figures S6 and S7).

#### 4.1.4 Controlled Synthesis of MoSe_2_–WSe_2_ HS I and HS II

A two‐zone furnace equipped with a quartz tube of 55 mm diameter (Carbolite Gero) was used for the growth in a similar manner as reported [43]. The growth substrate was heated slowly (15°C min^−1^) to different growth temperatures in the range of 770°C–900°C and maintained for 15 min to grow the HS I (at 1:3 and 1:10) and HS II (at 3:1).

#### 4.1.5 Transfer of Heterostructure on TEM grid

The HS II were transferred onto the lacey carbon‐coated Cu TEM grid via the wet transfer method [67]. In brief, a layer of poly(methyl methacrylate) (PMMA) was spin‐coated onto the as‐grown HS. Thereafter, the sample was floated on top of potassium hydroxide aqueous solution (2 M) to delaminate the PMMA/HS film from the SiO_2_/Si substrate and subsequently washed with ultrapure water to remove residual KOH. Afterwards, the PMMA/HS film was placed onto the TEM grid and baked at 90°C for 10 min to promote adhesion between the HS and lacey carbon on Cu. Next, the PMMA/HS/grid samples were introduced into a CO_2_ critical point dryer (CPD, Tousimis)(a similar description is available in [68]) to dissolve PMMA and to achieve a contamination‐free clean surface of the HS before introducing it into the electron microscope. For the CPD, the PMMA/HS/grid samples were kept in acetone for 45 min in the process chamber. The acetone was replaced by liquid CO_2_ at low temperature (∼−2°C), maintaining a pressure of 5 MPa. To get the supercritical CO_2_ condition, the temperature was raised to 38°C at a pressure of 10 MPa for 10 min. The HS/grid samples were taken out of the CPD chamber once the temperature and pressure reached ambient conditions. The transfer of HSs onto an Au Quantifoil TEM grid was performed without PMMA, using isopropanol and KOH as transfer agents.

### 4.2 Characterization

#### 4.2.1 Optical Microscopy

Axio Imager Z1.m microscope (Zeiss) equipped with a thermoelectrically cooled 3‐megapixel CCD camera (Axiocam 503 color) was used to capture the OM images in bright‐field operation.

#### 4.2.2 Atomic Force Microscopy

AFM measurements were performed with a Ntegra system (NT‐MDT) in tapping mode at ambient conditions using n‐doped silicon cantilevers (NSG01, NT‐MDT) with a tip radius of 10 nm with resonant frequencies of 167 kHz.

#### 4.2.3 Raman and PL Spectroscopy

Raman spectra were acquired using a Bruker Senterra spectrometer operated in backscattering mode. A 532 nm laser was used to excite the sample under a 100× objective, and a thermoelectrically cooled CCD detector collected the Raman signals through 1800 L/mm grating. The spectral resolution of the system is 2–3 cm^−1^. The Si peak at 521 cm^−1^ was used for calibration of the instrument. For PL spectra, the same 532 nm (2.33 eV) excitation was used with a grating of 1800 L/mm. Raman spectroscopy measurements were performed using a 2 mW excitation power and an integration time of 2 s. Raman and PL mapping were performed using a Renishaw inVia Raman microscope, operated in backscattering mode with a 532 nm excitation laser (Nd:YAG laser). A 100× objective lens with NA 0.85 was used for all measurements, and the system was equipped with an 1800 L/mm grating. The sample stage was motorized to enable automated mapping, and data were collected using a CCD detector. The acquired mapping data were analyzed using Renishaw's Wire 5.6 software.

#### 4.2.4 Scanning Electron Microscopy

SEM images were acquired using a Zeiss Sigma VP scanning electron microscope operated at an accelerating voltage of 2 kV, using the in‐lens detector.

#### 4.2.5 Transmission Electron Microscopy (HRTEM, STEM, and EDX mapping)

Prior to TEM analysis, the samples were removed from the substrate and transferred onto the TEM grids coated with lacey carbon films. HRTEM, high‐angle annular dark‐field scanning TEM (HAADF‐STEM) imaging, and energy dispersive x‐ray (EDX) mapping were acquired on a Thermo Fisher Talos 200× equipped with a SuperX four‐quadrant EDX detector. The microscope was either operated at 80 or 200 kV with a probe current of 100 to 200 pA in STEM mode and about 5 nA in TEM mode. An 80 kV accelerating voltage was used initially because of reduced knock‐on damage and higher inelastic scattering, improving the EDX sensitivity. However, at 200 kV, the sample contamination in STEM mode is reduced, and the spatial resolution in TEM and STEM mode is higher. The EDX energy dispersion was set to 5 eV per channel over an energy range of 0–20 keV. For EDX analysis, multiple drift‐corrected single‐frame scans (512 × 512 pixels) were taken and summed up. Typical mapping times were about 10 min; Owning to the low signal‐to‐noise ratio from ML regions, faint signals outside the flake originate from background noise only.

#### 4.2.6 Encapsulation of as‐Grown Samples

To provide the samples with a clean and strain‐free dielectric environment, they are encapsulated in layers of hexagonal boron nitride (hBN). A hot pick‐up technique [38] using polydimethylsiloxane (PDMS) and poly(bisphenol‐A) carbonate (PC) is employed for the encapsulation. Following the removal of the PC film, the encapsulated sample was annealed at 110°C for 20 min.

#### 4.2.7 Optical Spectroscopy of hBN Encapsulated Samples

For the optical spectroscopy carried out between *T* = 4 and 300 K, the samples were placed in an attoDRY800 ultra‐low vibration, closed‐cycle cryostat system, and a home‐built microscopy setup was used. The spectra were acquired using a Czerney‐Turner spectrograph equipped with a CCD Camera. For PL at *T* = 4 K, the excitation laser power was 20 µW, with an acquisition time of 20 ms. For the temperature‐dependent PL measurements, the laser power was kept constant at 20 µW, while the acquisition time varied between 20 and 5000 ms; all data were normalized to /s. A 533 nm excitation laser was used, with a laser spot size of approximately 580 nm. We used low‐temperature piezo‐positioners (attocube systems, ANPx101 and ANPz102) to position the sample and perform line scans. To achieve a diffraction‐limited excitation spot, a low‐temperature apochromatic objective was used. PL measurements were performed in backscattering geometry.

## Author Contributions

The manuscript was written through the contributions of all authors. All authors have given approval to the final version of the manuscript.

## Conflicts of Interest

The authors declare no conflicts of interest.

## Supporting information



It includes additional characterization of HS I and HS II heterostructures using OM, AFM, PL, and TEM, along with growth temperature profiles. Supporting tables provide precursor ratios, molar concentrations, Varshni parameters for ML MoSe_2_, and IX_T_ and IX_S_ features from HS II and exfoliated ML WSe_2_.
**Supporting File**: adma74172‐sup‐0001‐SuppMat.pdf.

## Data Availability

The data that support the findings of this study are available from the corresponding author upon reasonable request.
