# Supplementary material for: CVD Grown Hybrid MoSe2–WSe2 Lateral/Vertical Heterostructures With Strong Interlayer Exciton Emission
Source: Adv Mater. 2026 Jul 25;38(57):e74172. doi: 10.1002/adma.74172 (PMC13660820; doi:10.1002/adma.74172)
Supplement: Supplementary file 1 [file ADMA-38-e74172-s001.pdf]

Supporting Information

CVD Grown Hybrid $MoSe_2$-WSe_2 Lateral/Vertical Heterostructures with Strong Interlayer Exciton Emission

Md Tarik Hossain[1], Sai Shradha[2], Axel Printschler[1], Julian Picker[1], Luc F. Oswald[2], Julian Führer[2], Nicole Engel[2], Honey Jayeshkumar Shah[1], Christof Neumann[1], Daria I. Markina[2], Moritz Quincke[3], Johannes Biskupek[3], Kenji Watanabe[4], Takashi Taniguchi[5], Ute Kaiser[3], Bernhard Urbaszek[2], Andrey Turchanin[1,6]**

[1]Institute of Physical Chemistry, Friedrich Schiller University Jena,
Lessingstr. 10, 07743 Jena, Germany

[2]Institute of Condensed Matter Physics, Technical University Darmstadt,
Hochschulstraße 6-8, 64289 Darmstadt, Germany

[3]Central Facility of Electron Microscopy, Electron Microscopy Group of Material Science, Ulm University, 89081 Ulm, Germany

[4]Research Center for Electronic and Optical Materials,
National Institute for Materials Science, 1-1 Namiki, Tsukuba 305-0044, Japan

[5]Research Center for Materials Nanoarchitectonics,
National Institute for Materials Science, 1-1 Namiki, Tsukuba 305-0044, Japan

[6]Abbe Center of Photonics, Albert-Einstein-Straße 6, 07745 Jena, Germany

Corresponding Authors:

Md Tarik Hossain (tarik.hossain@uni-jena.de),

Andrey Turchanin (andrey.turchanin@uni-jena.de)

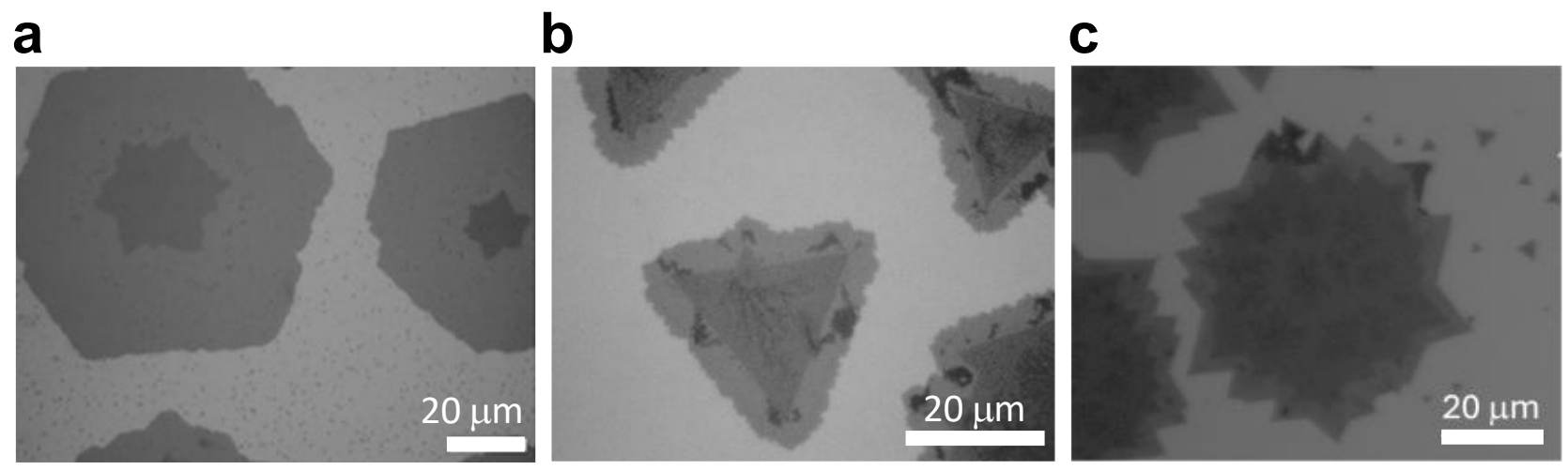

Figure S1: Optical microscopy images of differently shaped lateral $MoSe_2$-WSe_2 HS I. (a) hexagonal, (b) triangle, (c) jagged-edged.

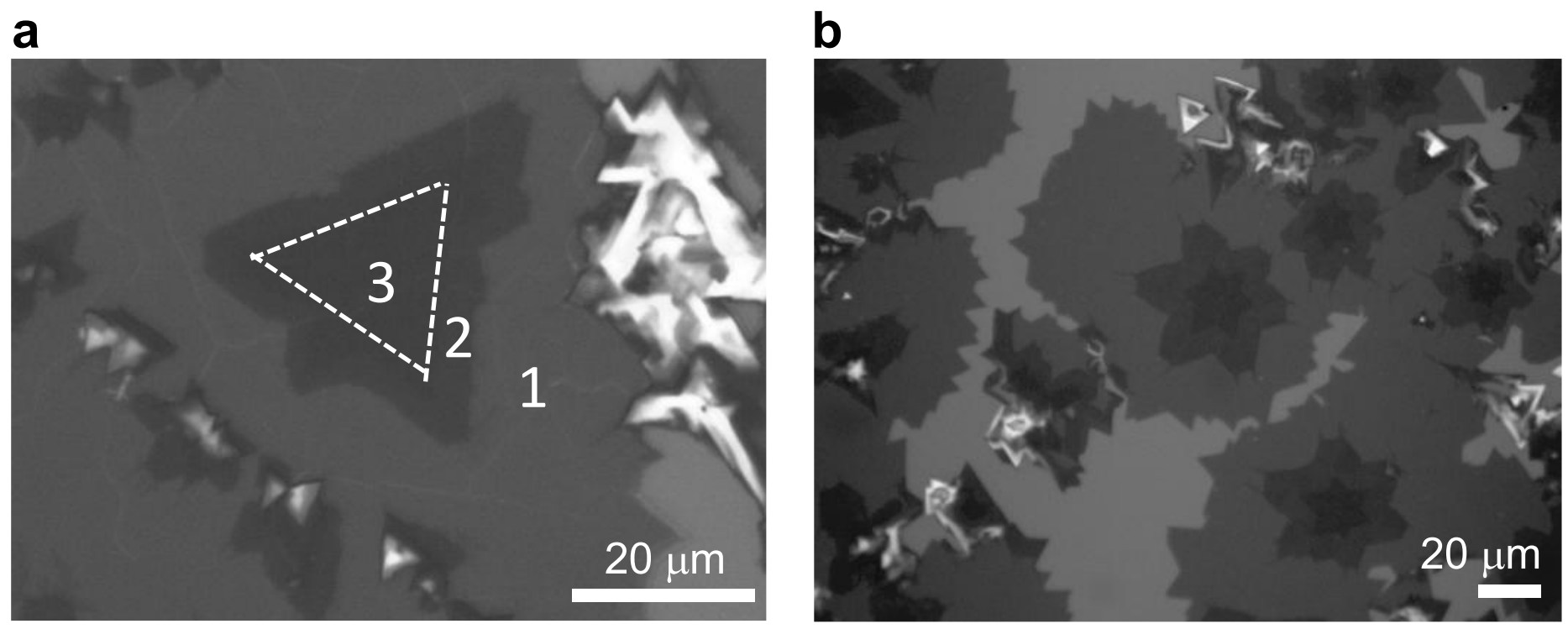

Figure S2: Optical microscopy images of a differently shaped $MoSe_2$-WSe_2 HS II (lateral/vertical heterostructure). (a) triangle, (b) hexagon on the bottom jagged-edged $MoSe_2$ monolayer.

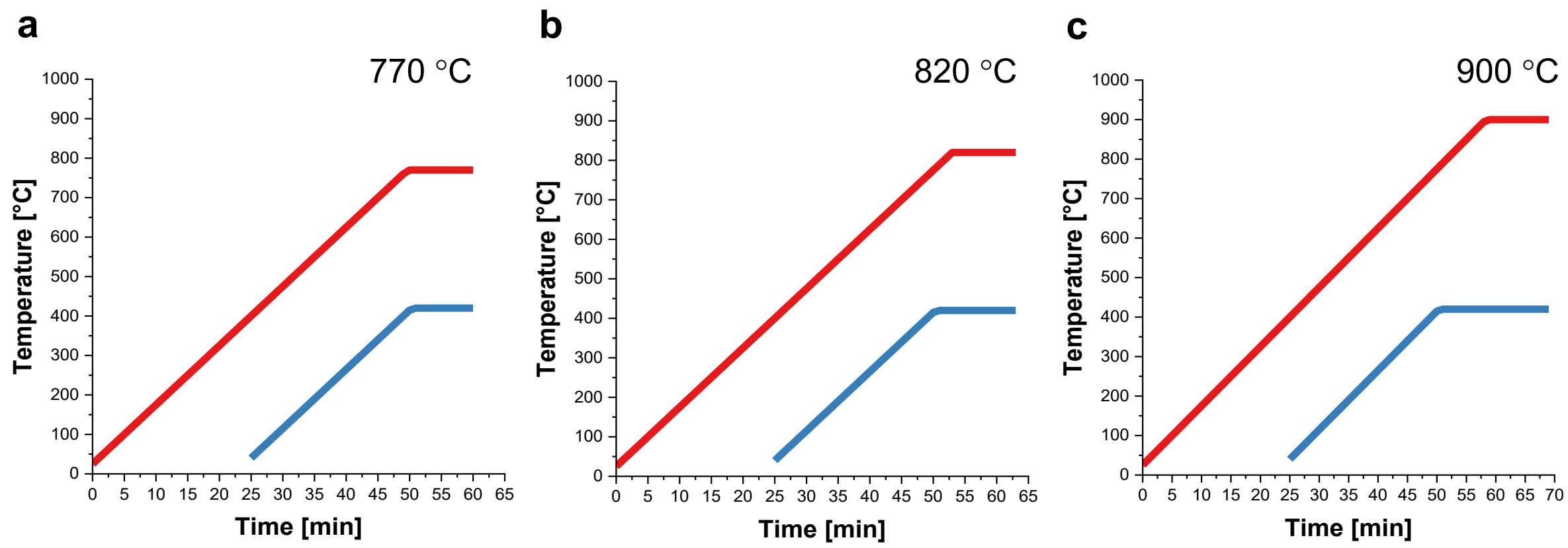

Figure S3: CVD temperature profiles for 770 °C (a), 820 °C (b), 900 °C (c). The red curves represent the heating profiles of the transition metal precursors, while the blue curves correspond to the heating of the selenium precursor.

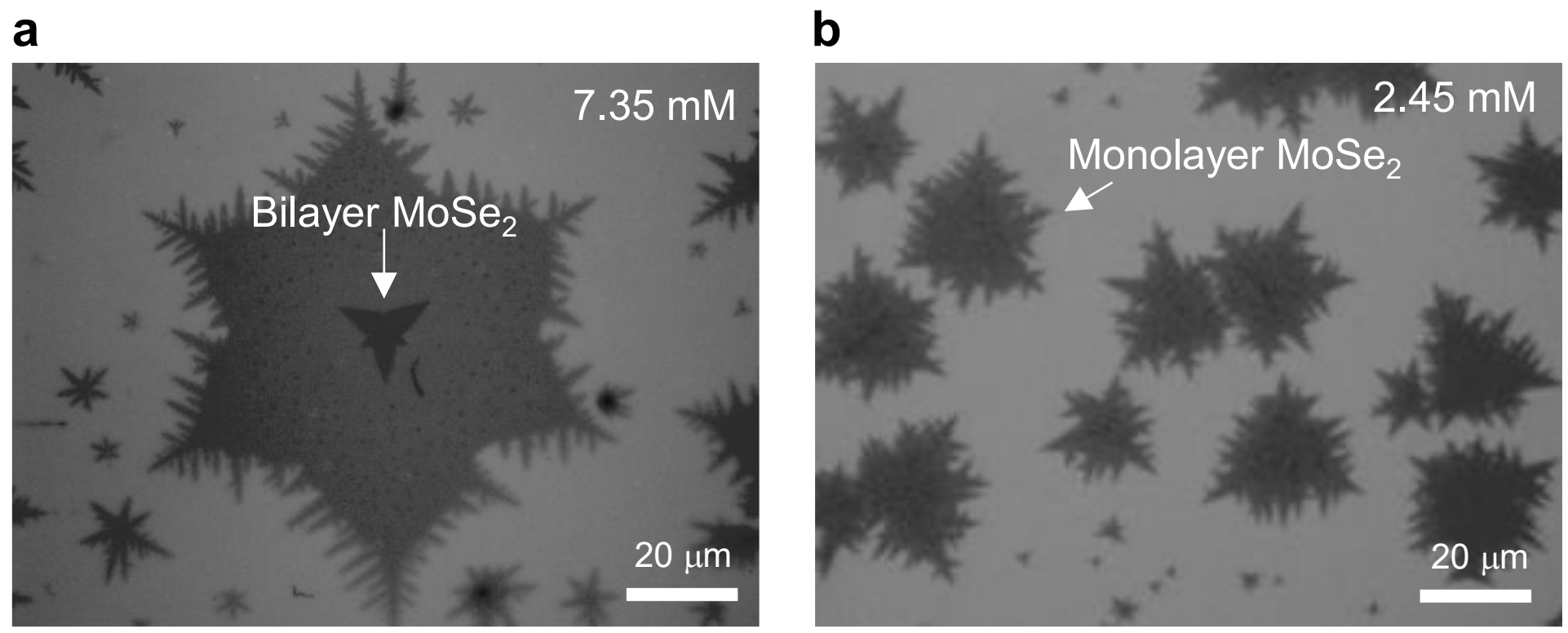

Figure S4: Optical microscopy image of bilayer (2L) (a) and monolayer (b) $MoSe_2$ grown using 7.35 mM and 2.45 mM of Na_2MoO_4 aqueous solution, respectively.

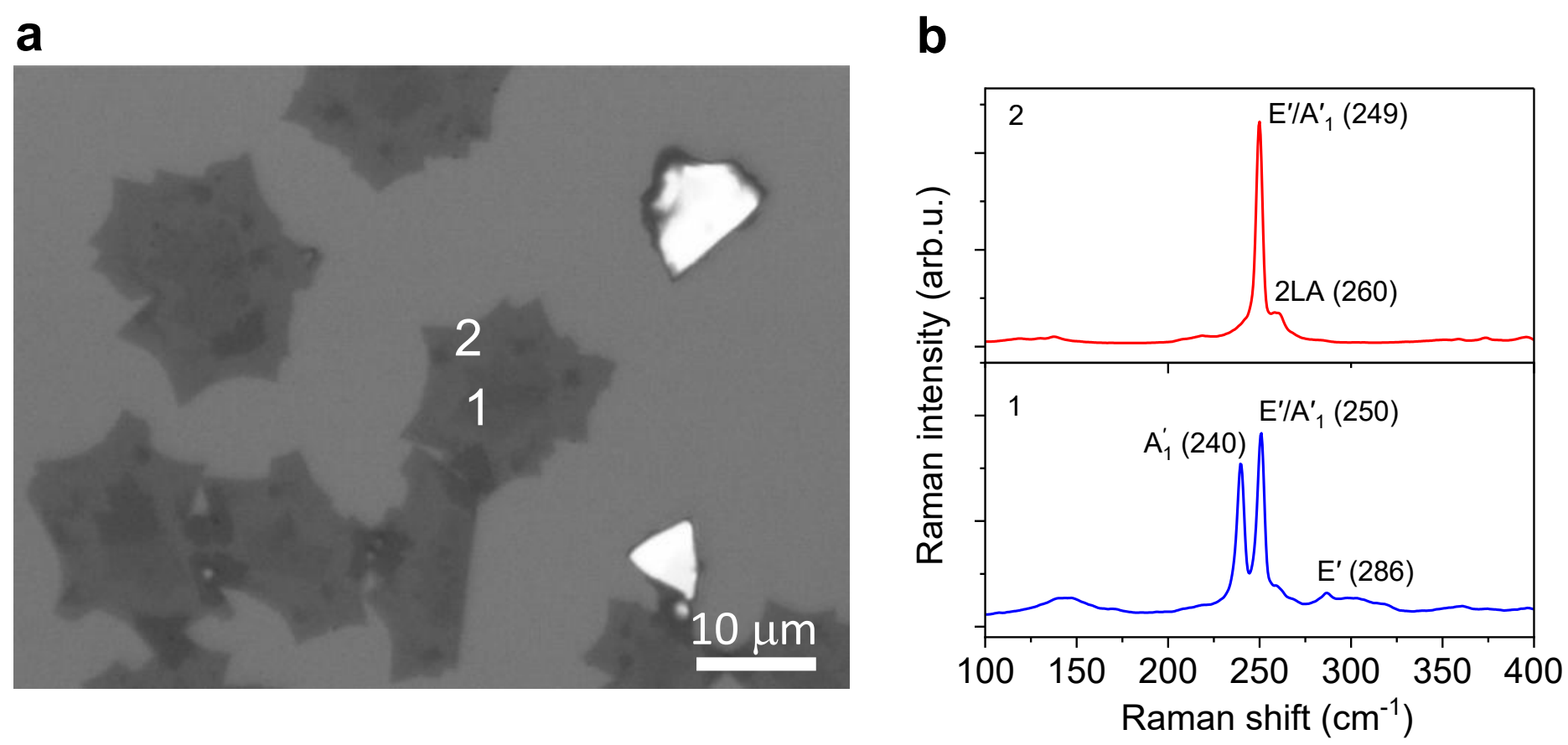

Figure S5: (a) Optical microscopy image of a mixed-phase lateral HS, and (b) corresponding Raman spectra collected from the inner region (1) and outer region (2). Region 1 contains both $MoSe_2$ and WSe_2 phases, while Region 2 shows only the WSe_2 phase.

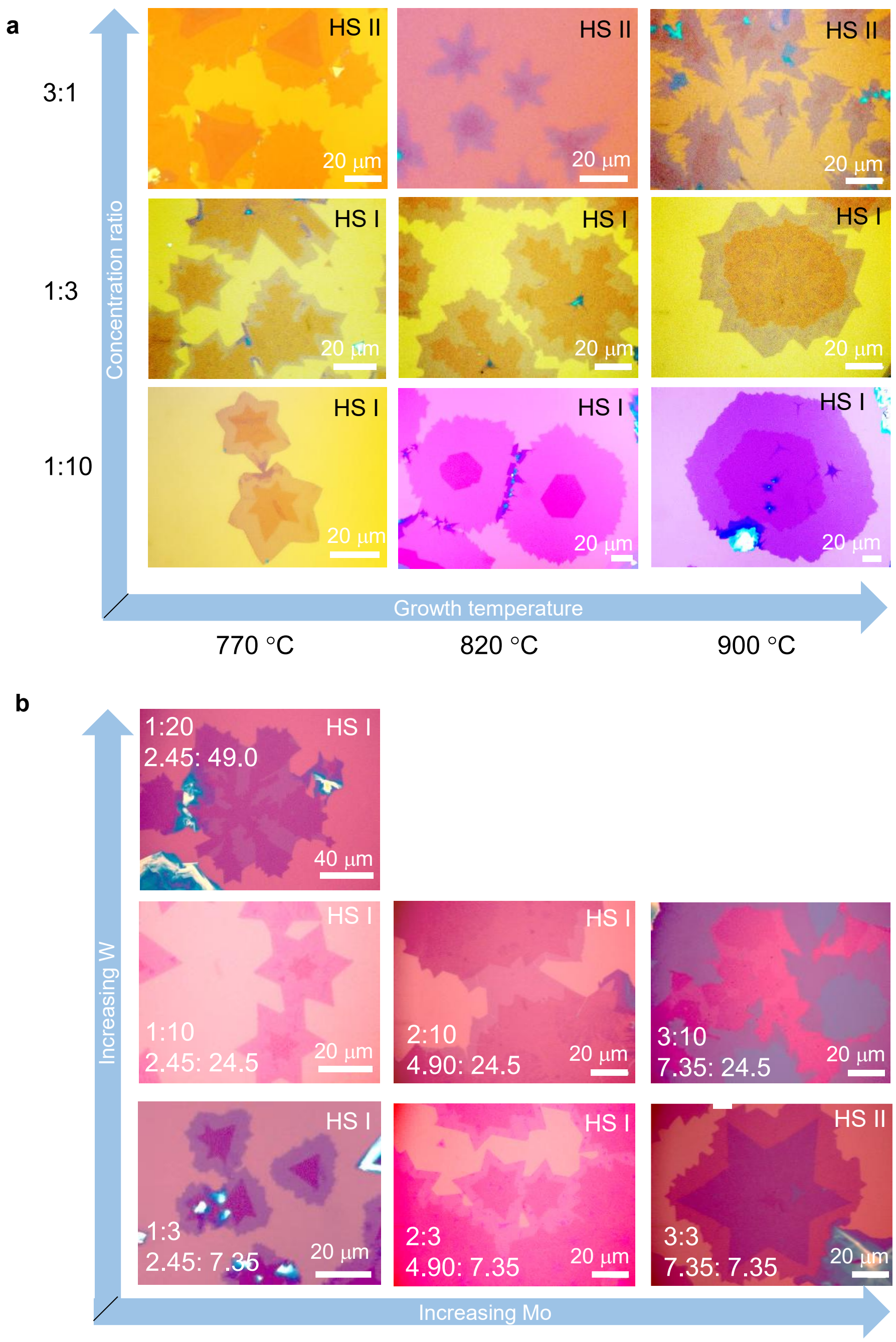

Figure S6: False color optical microscopy images of HS I and HS II as a function of growth temperature and precursor concentration ratio, showing the dependence of crystal size and morphology on synthesis conditions. Detailed analysis of crystal size is demonstrated in **Figure S7.** Note that the growth temperature in (b) is 820 °C.

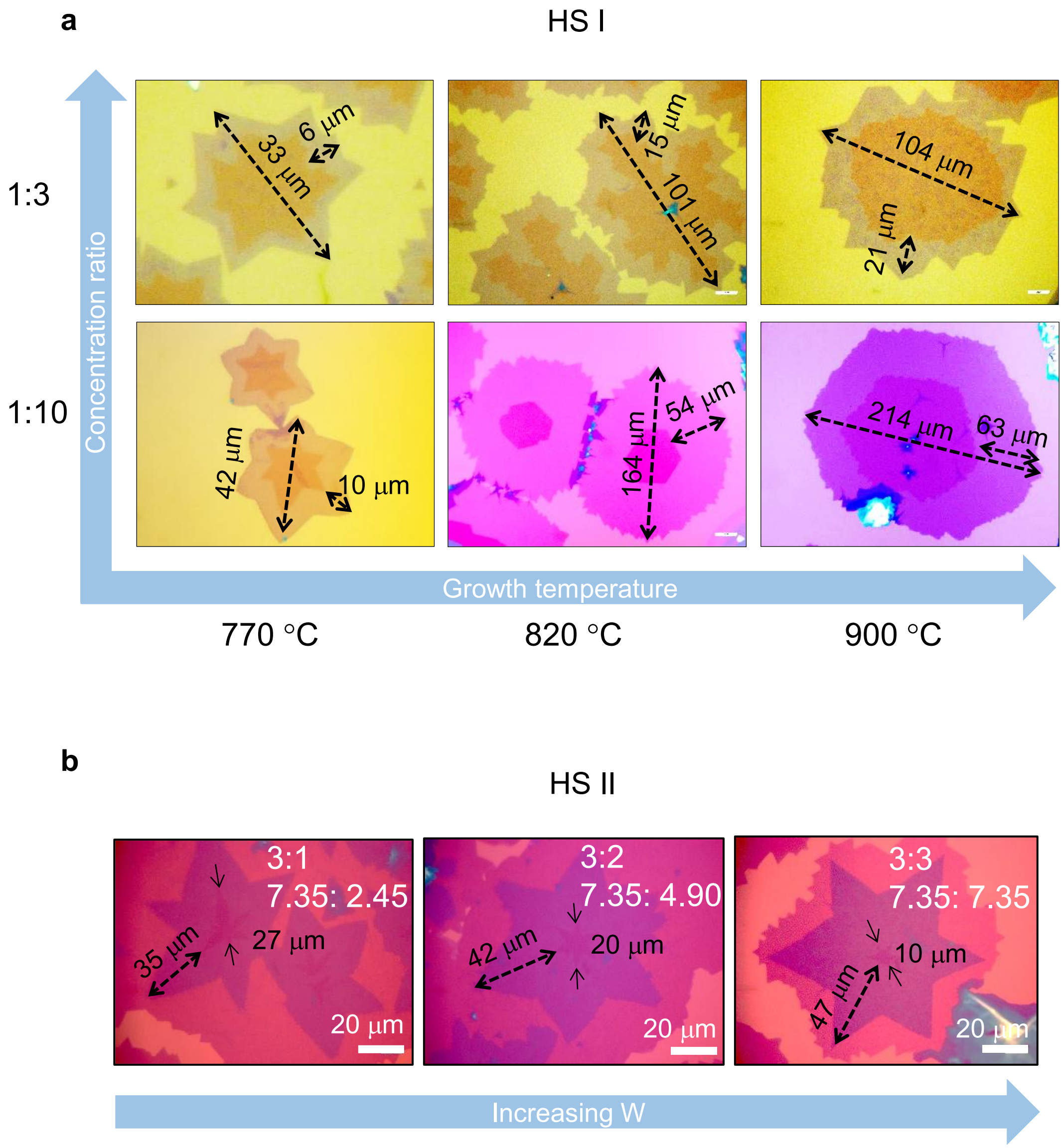

Figure S7: (a) False color optical images of HS I as a function of growth temperature and precursor concentration ratio, showing the dependence of overall crystal size and width of WSe_2 on synthesis conditions. The scale bar is identical to that in **Figure S6**. (b) False color optical images of HS II grown at 820 °C with different precursor concentration ratios, showing the dependence of $MoSe_2$ and WSe_2 domain width on synthesis conditions.

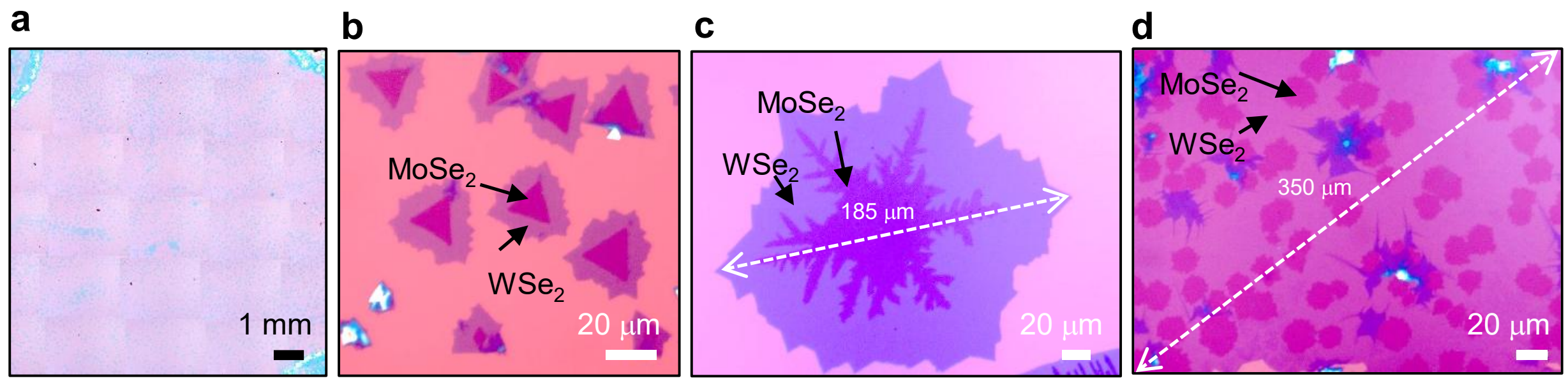

Figure S8: (a) HS I is grown uniformly across the entire substrate (12 × 12 mm^2). demonstrating the scalability of HS I growth using liquid precursors. Enlarged image (b) of isolated HS I (~10-50 μm) at the center (c) big isolated HS I (~185 μm) closer to the edge (d) continuous HS I (~350 μm) at the edge of the growth substrate.

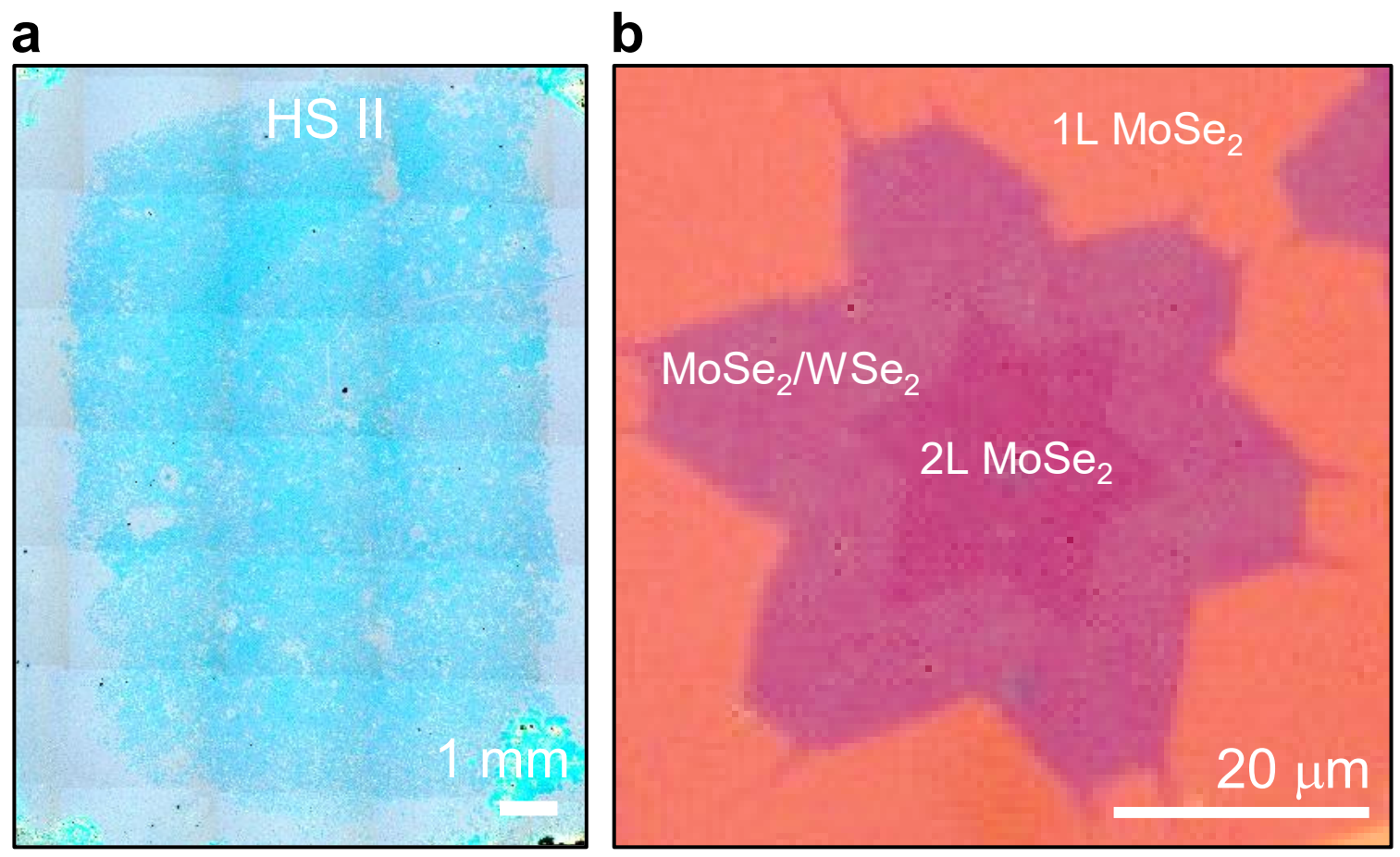

Figure S9: (a) HS II is grown uniformly across the substrate (8 × 12 mm^2), demonstrating the scalability of HS II growth using liquid precursors. (b) enlarged image of a HS II. Note that the blocky appearance in (a) is a stitching artifact.

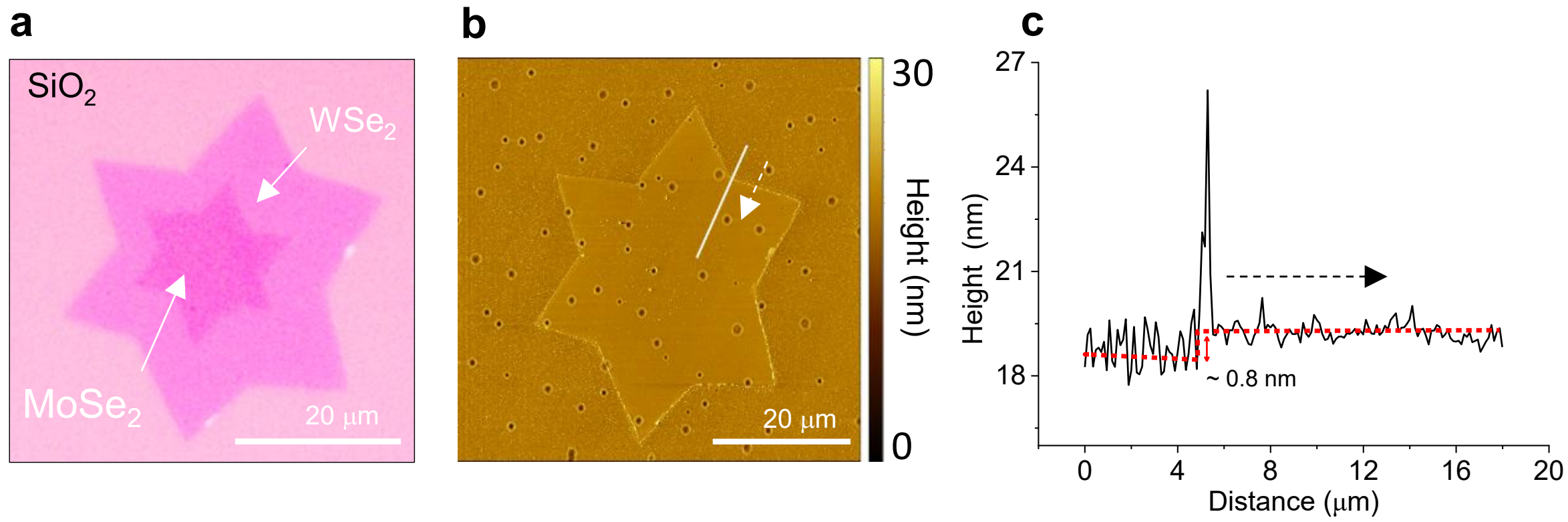

Figure S10: Microscopic characterization of HS I. (a) False color optical microscopy image of $MoSe_2$-WSe_2 HS I. (b) The corresponding topography AFM image. (c) shows the height profile along the white line between the SiO_2 substrate and the heterostructure. The thickness of the lateral heterostructure is estimated at ~ 0.8 nm, corresponding to a monolayer. Additionally, in the AFM images, the presence of black circular spots across the lateral HS and throughout the substrate can be recognised. This likely arises from precursor segregation (Figure S11), and the formation of Na_2O as a byproduct during annealing[1-3], which can react with SiO_2 to form Na_4SiO_4[4], leading to the formation of etch pits[5]. Despite this, Raman mapping (Figure 2b, c) and TEM analyses (Figure S12, 3a) confirm a continuous, high-quality $MoSe_2$-WSe_2 lateral HS.

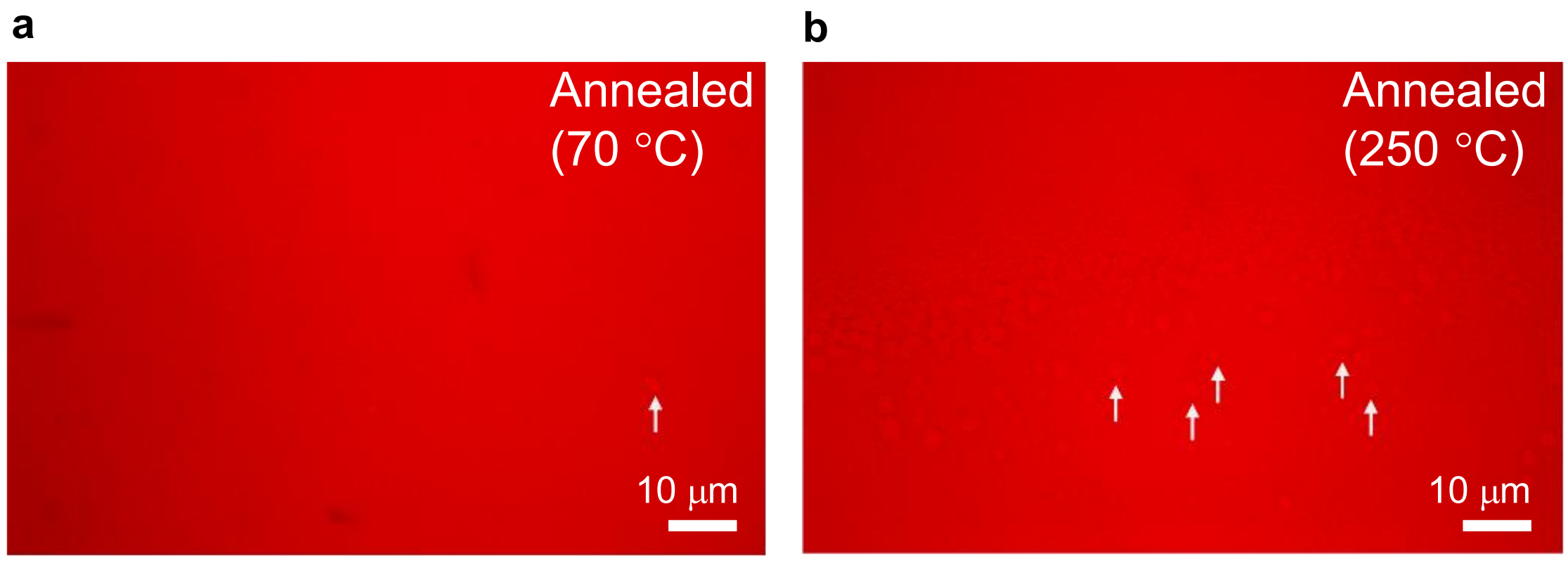

Figure S11: False color optical microscopy images after heating of deposited liquid precursor, which forms circular patterns. (a) at 70 °C and (b) 250 °C.

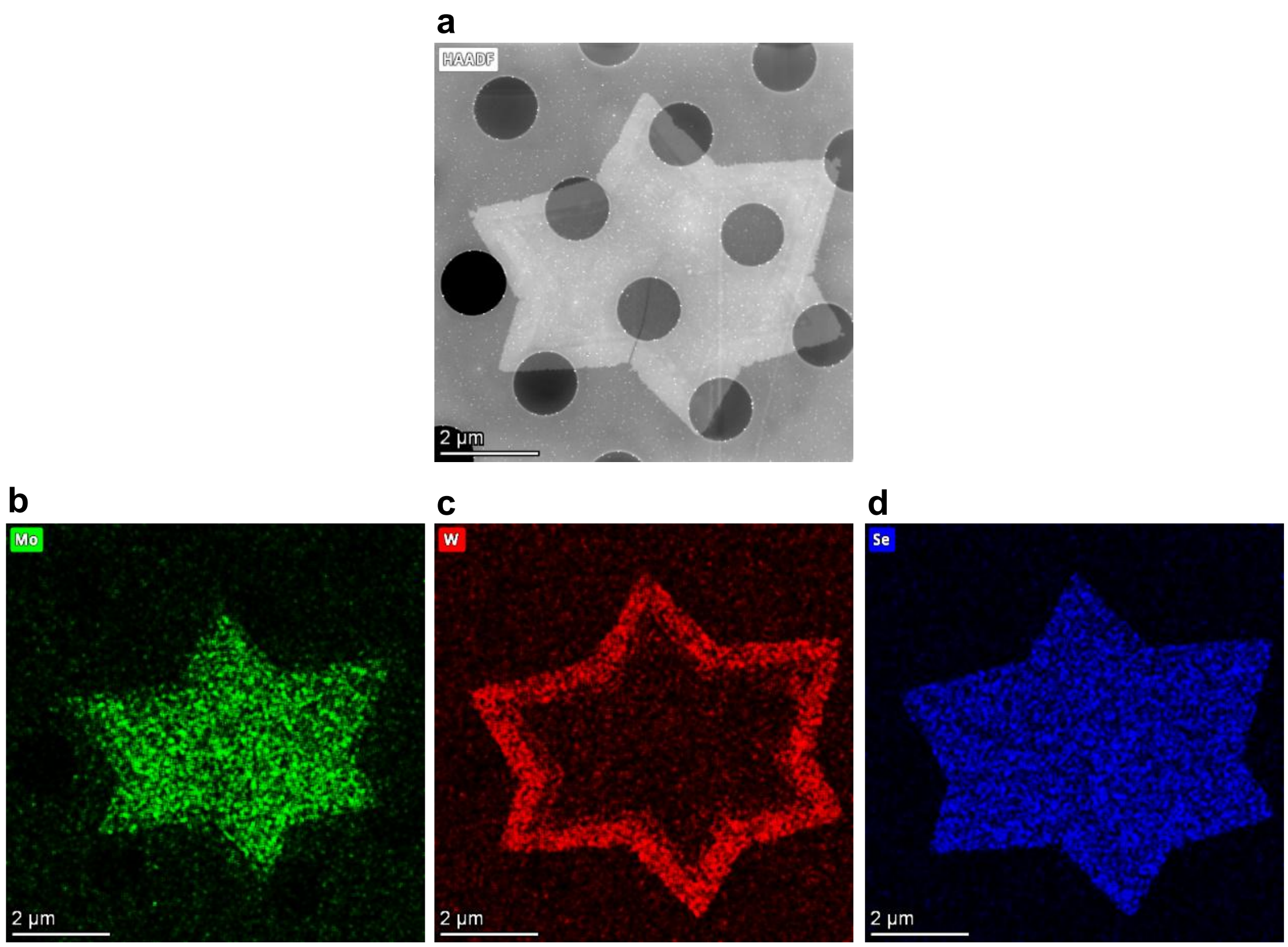

Figure S12: (a) 200 kV-HAADF-STEM image of the heterostructure. (b-d) EDX elemental maps representing the spatial distribution of Se (blue), Mo (green), and W (red).

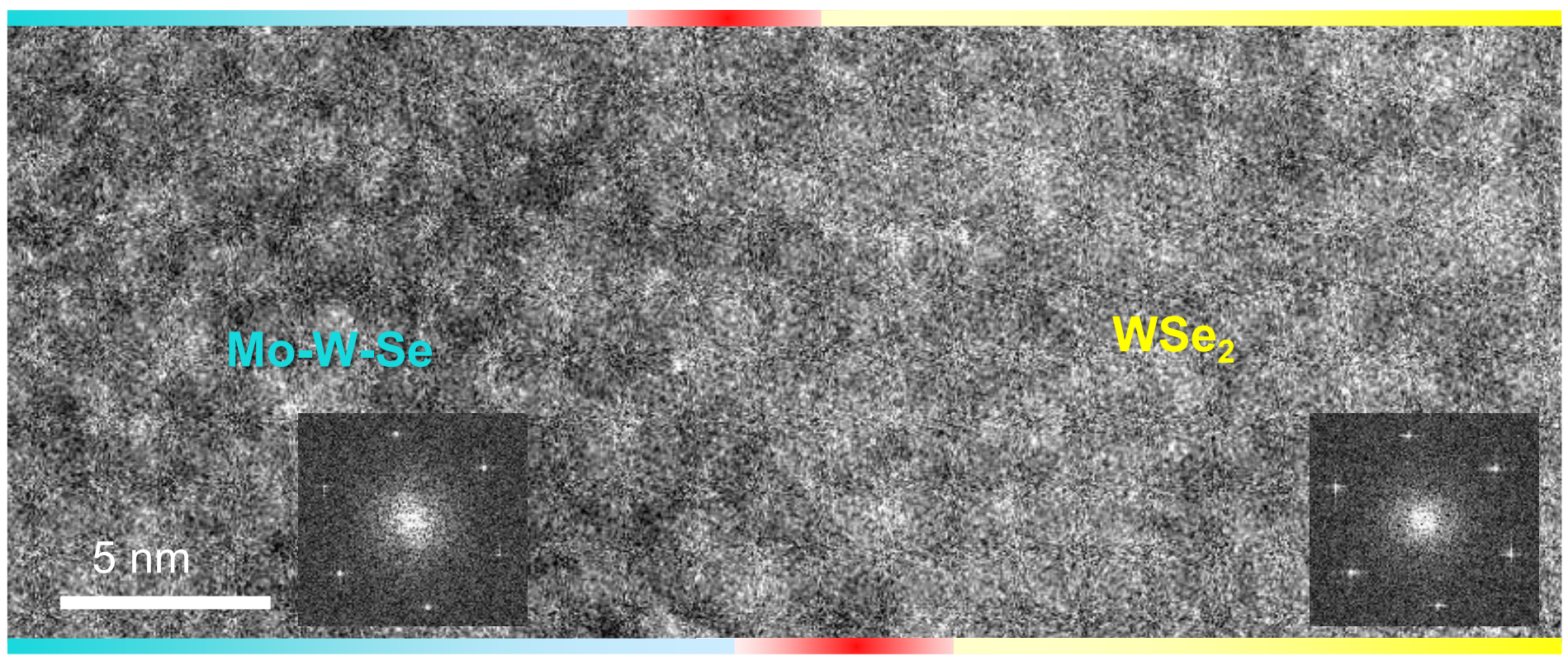

Figure S13: (a) High-magnification 200 kV HAADF-STEM image of the Mo-W-Se and WSe_2 boundary shows a narrow ≈ 5 nm interface (red lines), with Mo-W-Se and WSe_2 regions indicated by cyan and yellow lines, respectively. The inset displays the corresponding fast Fourier transforms (FFTs) obtained from the respective regions.

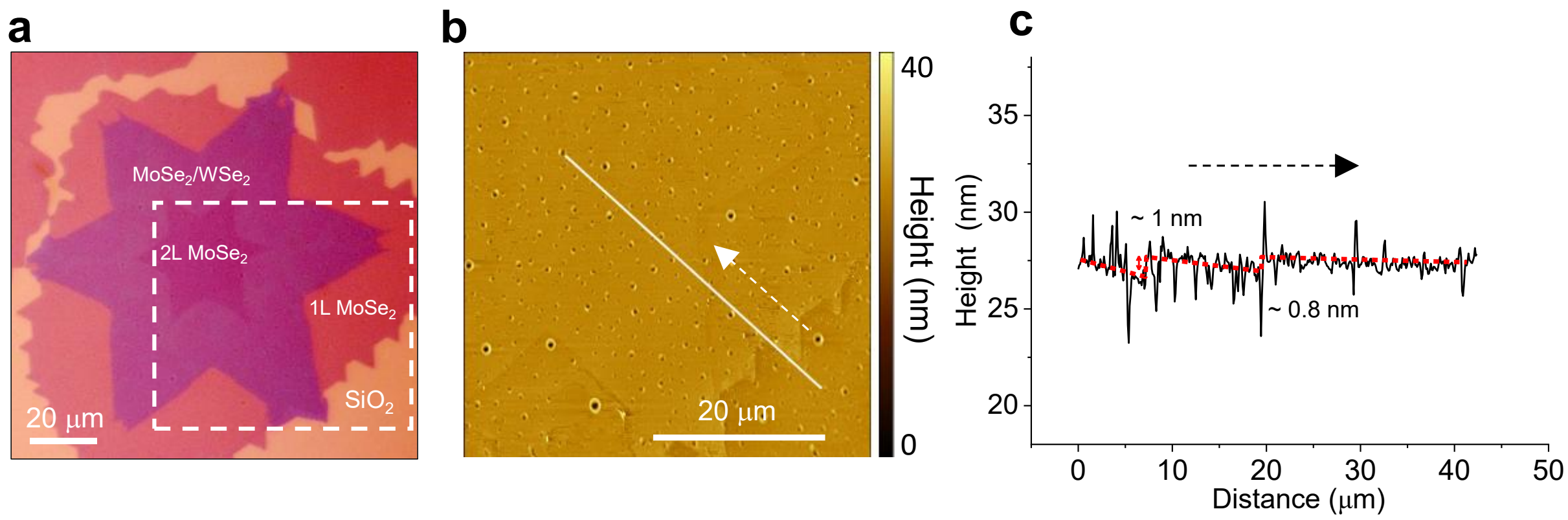

Figure S14: Microscopic characterization of HS II. (a) False-color optical microscopy image of $MoSe_2$-WSe_2 HS II. (b) represents an AFM topography image in the area highlighted within the dashed square in (a). The topography image shows height contrast between the SiO_2 substrate and the bottom $MoSe_2$ layer, as well as between the bottom $MoSe_2$ layer and the top lateral heterostructure. (c) shows the height profile extracted along the solid white line in (b), highlighting the step edges across different regions. The step height of ~1 nm between SiO_2 and $MoSe_2$ corresponds to a monolayer (1L) of $MoSe_2$. The additional step of ~ 0.8 nm from $MoSe_2$ to the top WSe_2 indicates the formation of a second monolayer, resulting in a bilayer heterostructure. Notably, no distinguishable height difference is observed at the $MoSe_2$-WSe_2 lateral junction, which further confirms the formation of the expected ML lateral HS on the bottom $MoSe_2$ layer. The spikes observed in the height profile originate from the formation of etch pits, as discussed in Figure S10.

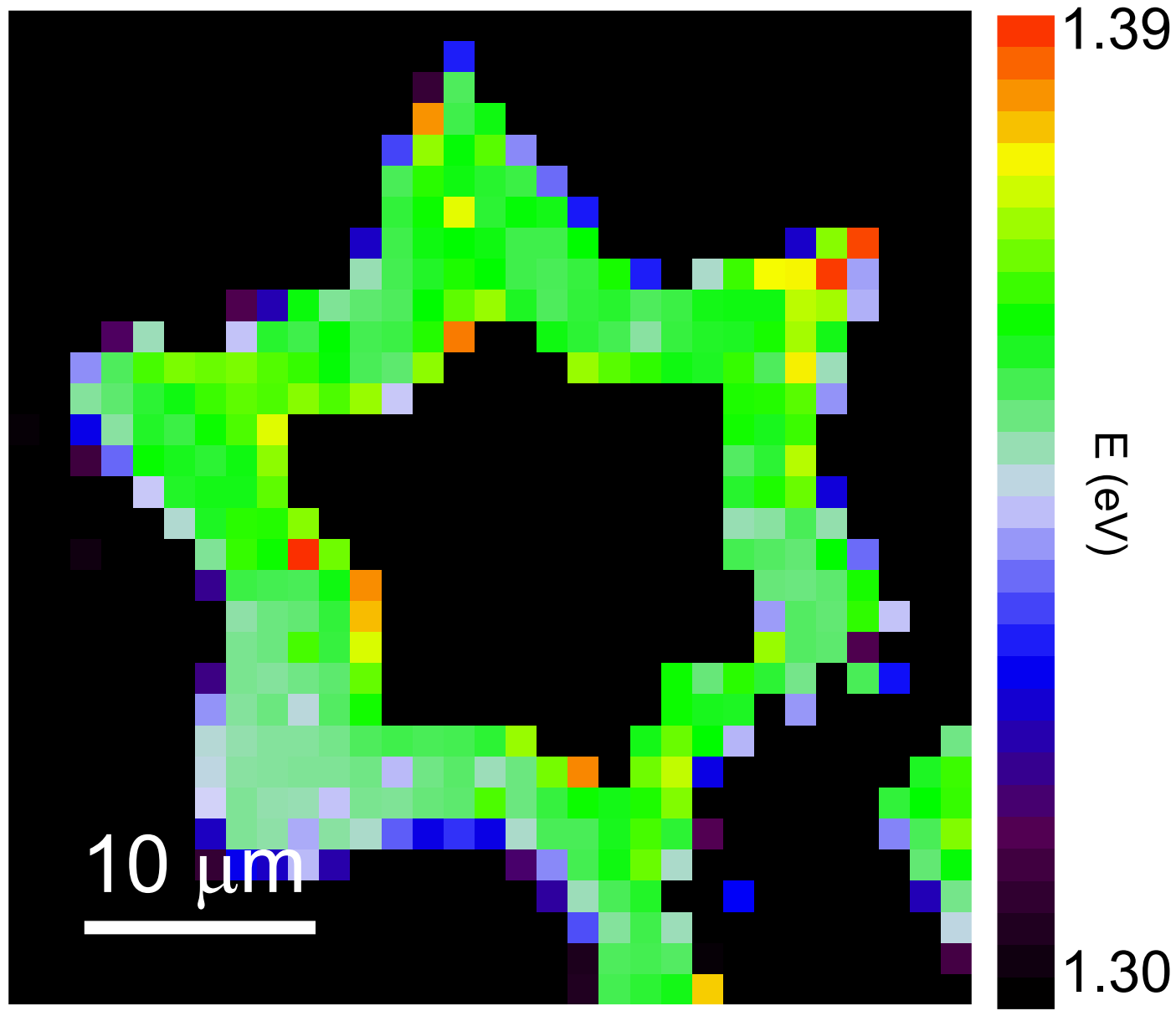

Figure S15. Representative interlayer exciton (IX) emission energy map at room temperature of $MoSe_2/WSe_2$ heterobilayer in HS II. The map shows the spatial distribution of the IX peak position within the energy range of 1.30 - 1.39 eV.

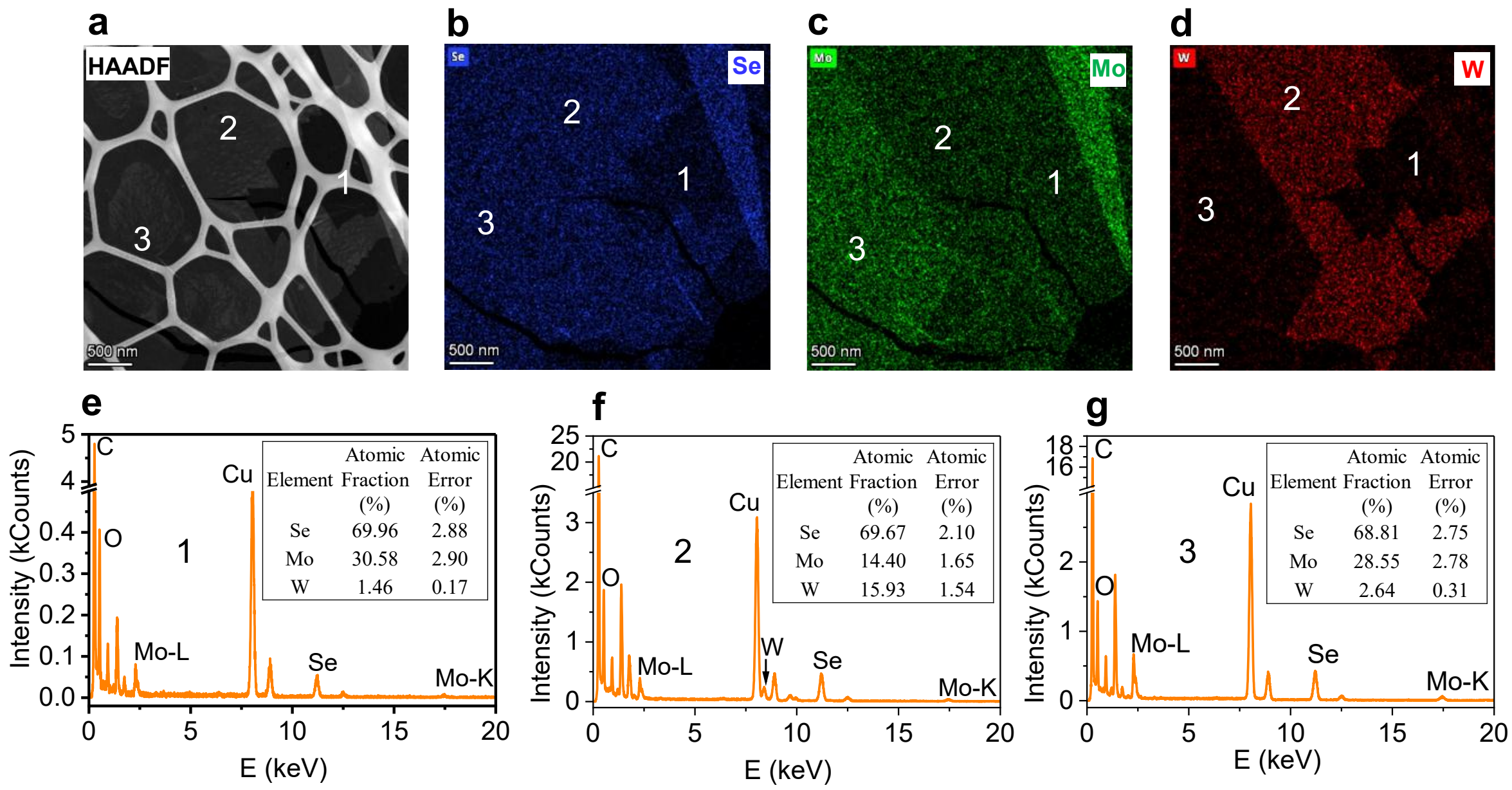

Figure S16: (a) 80 kV HAADF-STEM image of a HS II. (b-c) EDX elemental maps representing spatial distribution of Se (blue), Mo (green), and W (red). (e-g) Corresponding EDS spectra, with insets showing the atomic percentages of Se, Mo, and W at regions 1 (1L $MoSe_2$), 2 ($MoSe_2/WSe_2$ heterobilayer), and 3 (2L $MoSe_2$), respectively.

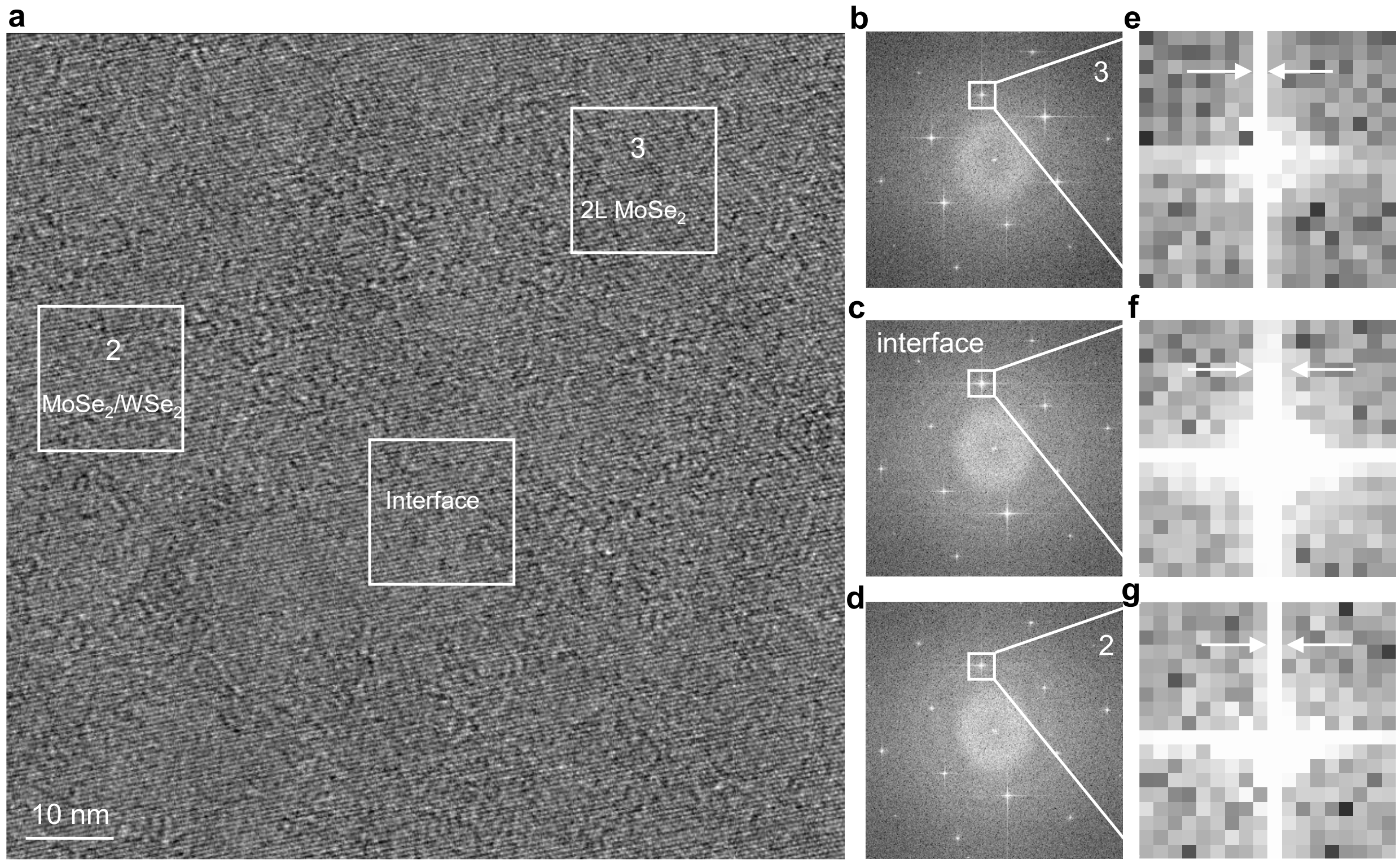

Figure S17: (a) 200 kV High-resolution TEM (HRTEM) image of the top lateral heterostructure in HS II showing continuous atomic rows across the top layer, indicative of epitaxial growth of the top LH. (b-d) Fast Fourier transform (FFT) patterns obtained from three different regions ($MoSe_2$ homobilayer, interface of top LH and $MoSe_2/WSe_2$ heterobilayer). (e-g) Corresponding magnified images of single diffraction spots highlight the transition from the homobilayer $MoSe_2$ (region 3) to the heterobilayer $MoSe_2/WSe_2$ (region 2). While regions 2 and 3 exhibit narrower fringes, the interface displays a wider fringe. The fringe shift between regions 2 and 3 supports the transitional boundary from homobilayer to heterobilayer. Please note that remaining amorphous and graphitic surface contaminations of residual hydrocarbons as results of the transfer process are overlaying the lattice structure of the TMDs and lowering the quality of the HRTEM images.

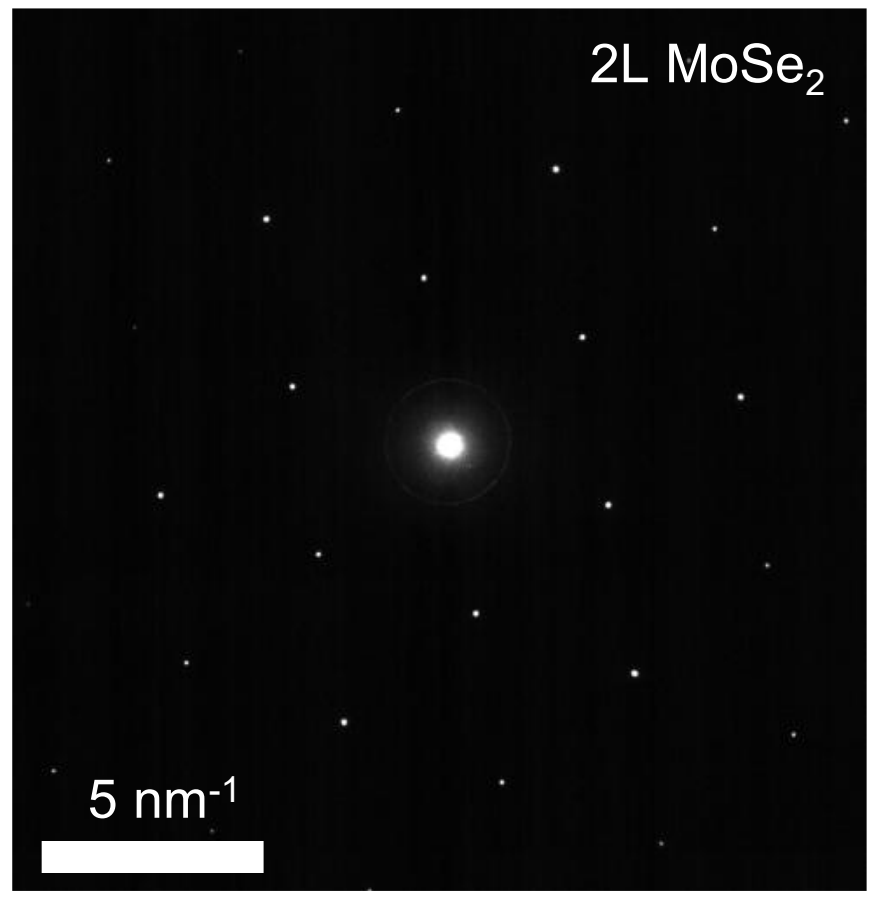

Figure S18: Selected-area electron diffraction (SAED) patterns acquired at 200 kV of bilayer (2L) $MoSe_2$ region of HS II display six-fold symmetry, characteristic of the hexagonal lattice. The presence of hexagonal diffraction pattern with circular spots supports the epitaxial growth of the top $MoSe_2$ ML.

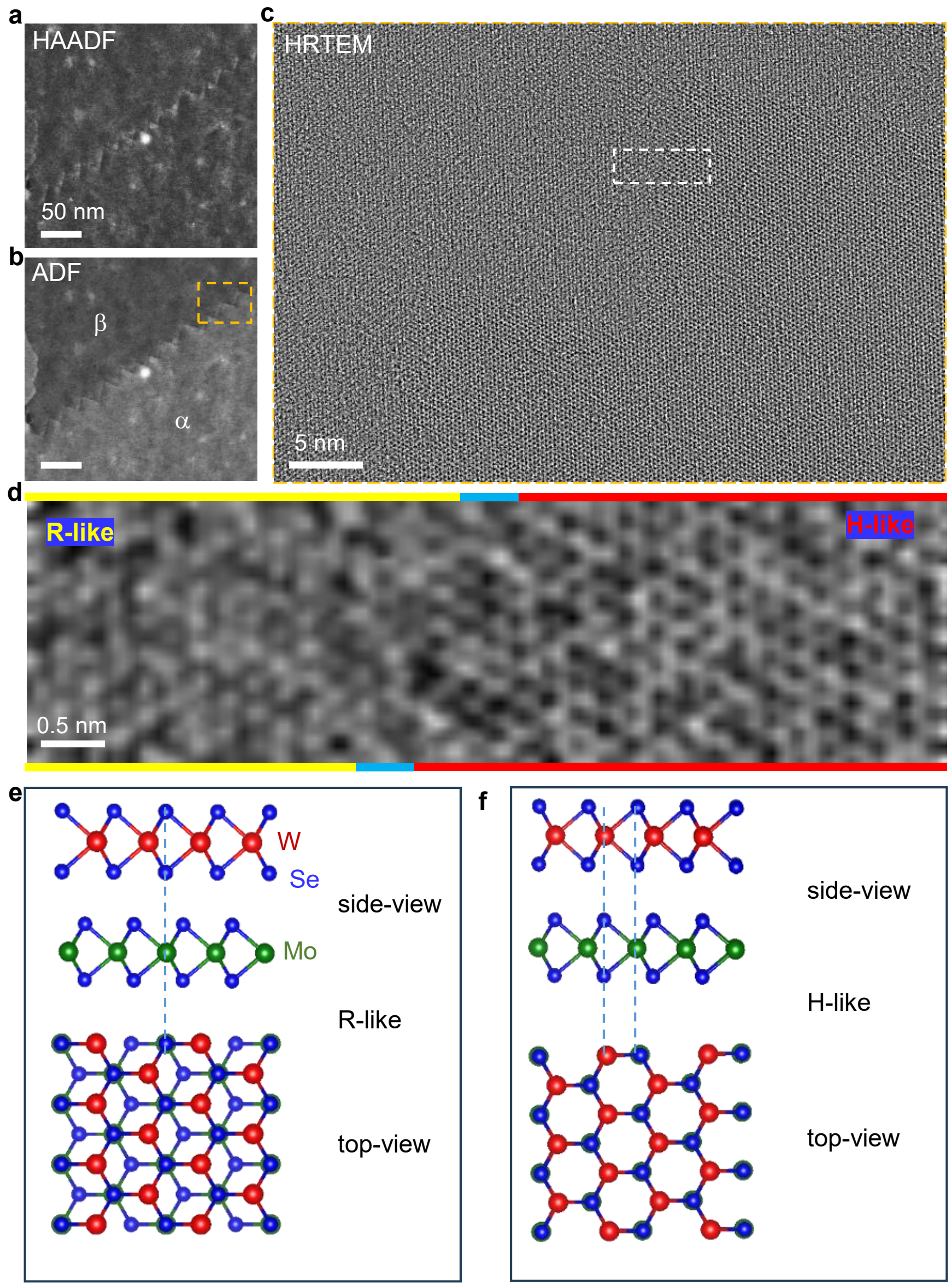

Figure S19: $MoSe_2/WSe_2$ heterobilayer with H and R-like stacking. (a,b) Comparison of 200 kV HAADF- and ADF-STEM images highlighting two domains (α and β, also denoted in main *figure 5h*), which are clearly resolved by brighter and darker contrast in the ADF image. (c) 200 kV high-resolution TEM image of the region marked by the orange dashed box in (b). (d) Magnified view of the area outlined by the white dashed box in (c), showing regions with AA (R-like) and AB (H-like) stacking. (e,f) Corresponding atomic models illustrating the stacking arrangements. In the AA (R-like) stacking, Se atoms of WSe_2 align above Mo atoms of $MoSe_2$, whereas in the AB (H-like) stacking, W (Se) atoms of WSe_2 are positioned above Se (Mo) atoms of the $MoSe_2$ layer, thereby forming a hollow hexagonal structure when viewed from the c-axis direction (top view).

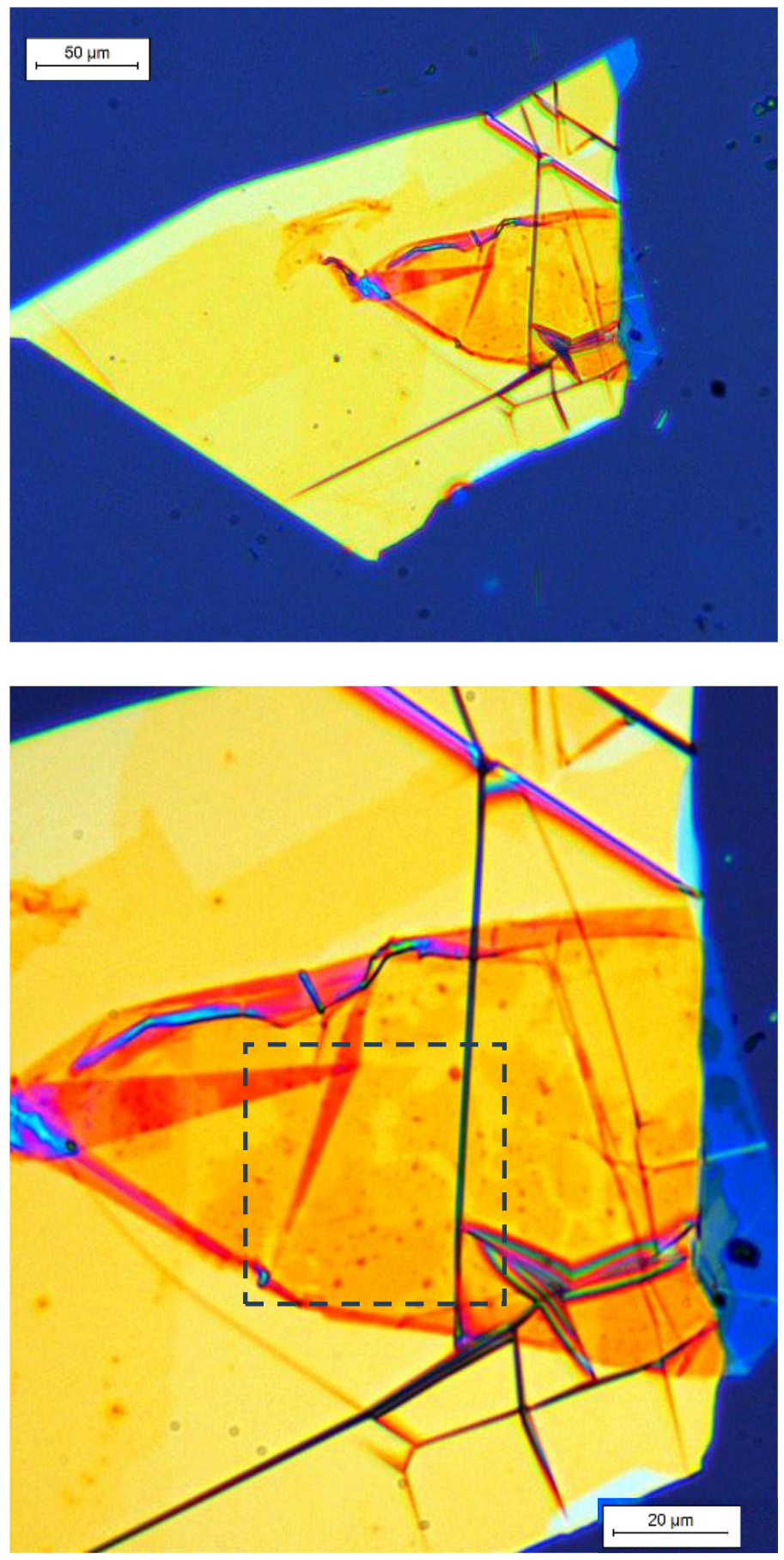

Figure S20: Optical microscope images of the hBN encapsulated HS II.

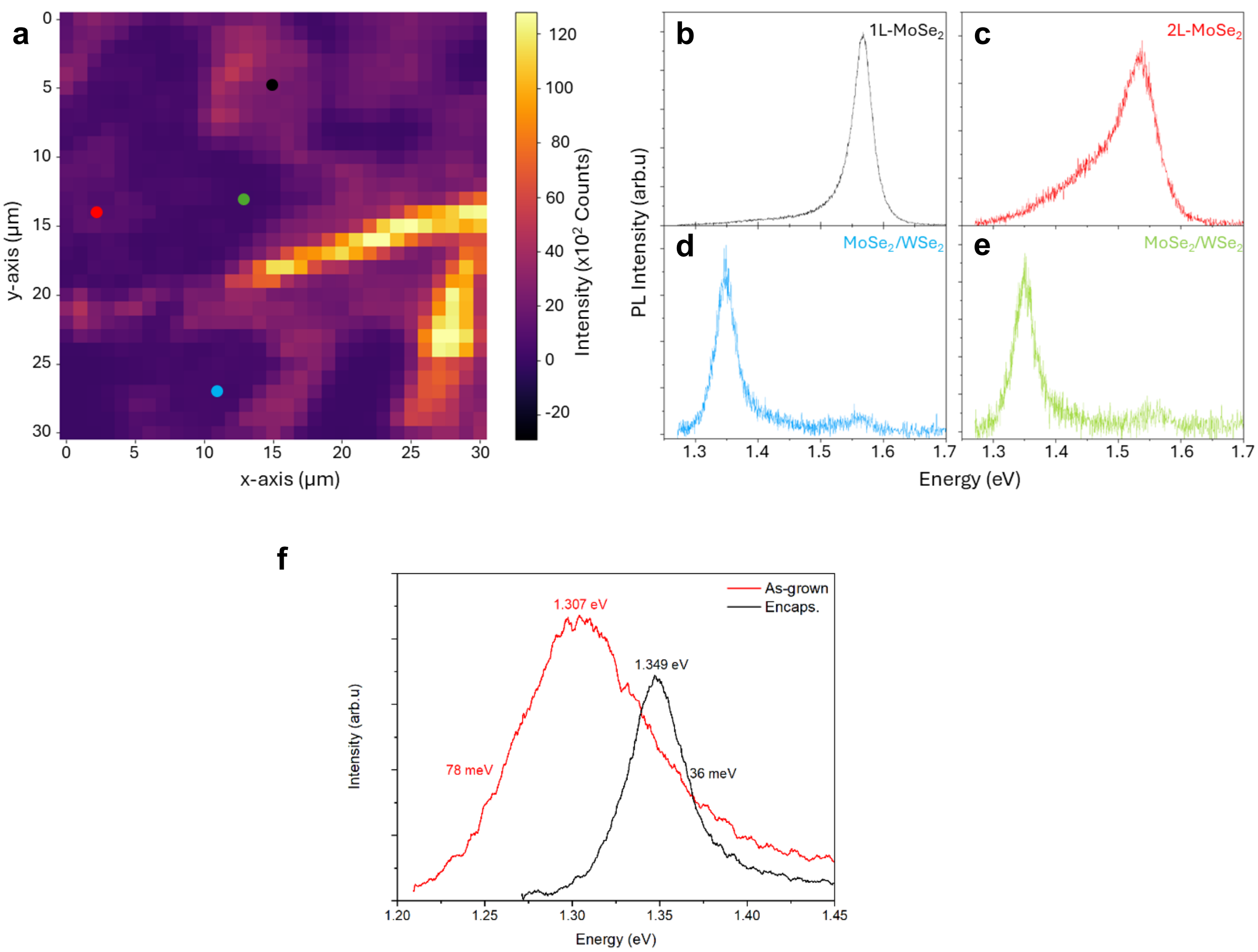

Figure S21: (a-e) Room temperature (T = 300K) PL data of hBN encapsulated HS II. PL map of encapsulated HS II (a). Spectra corresponding to the regions marked with colored dots are presented in b-e. PL spectra from (b) 1L-$MoSe_2$ (c) 2L-$MoSe_2$ and (d-e) $MoSe_2$-WSe_2 heterobilayer regions. (f) Room temperature IX emission from the as-grown and encapsulated samples. A redshift of 42 meV is observed in the as-grown sample, which likely arises due to the presence of intrinsic strain in the flakes. The emission linewidth narrows from 78 meV in the as-grown sample to 36 meV after hBN encapsulation. These observations demonstrate that hBN encapsulation provides a clean and more strain-free dielectric environment for optical spectroscopy on CVD-grown heterostructures.

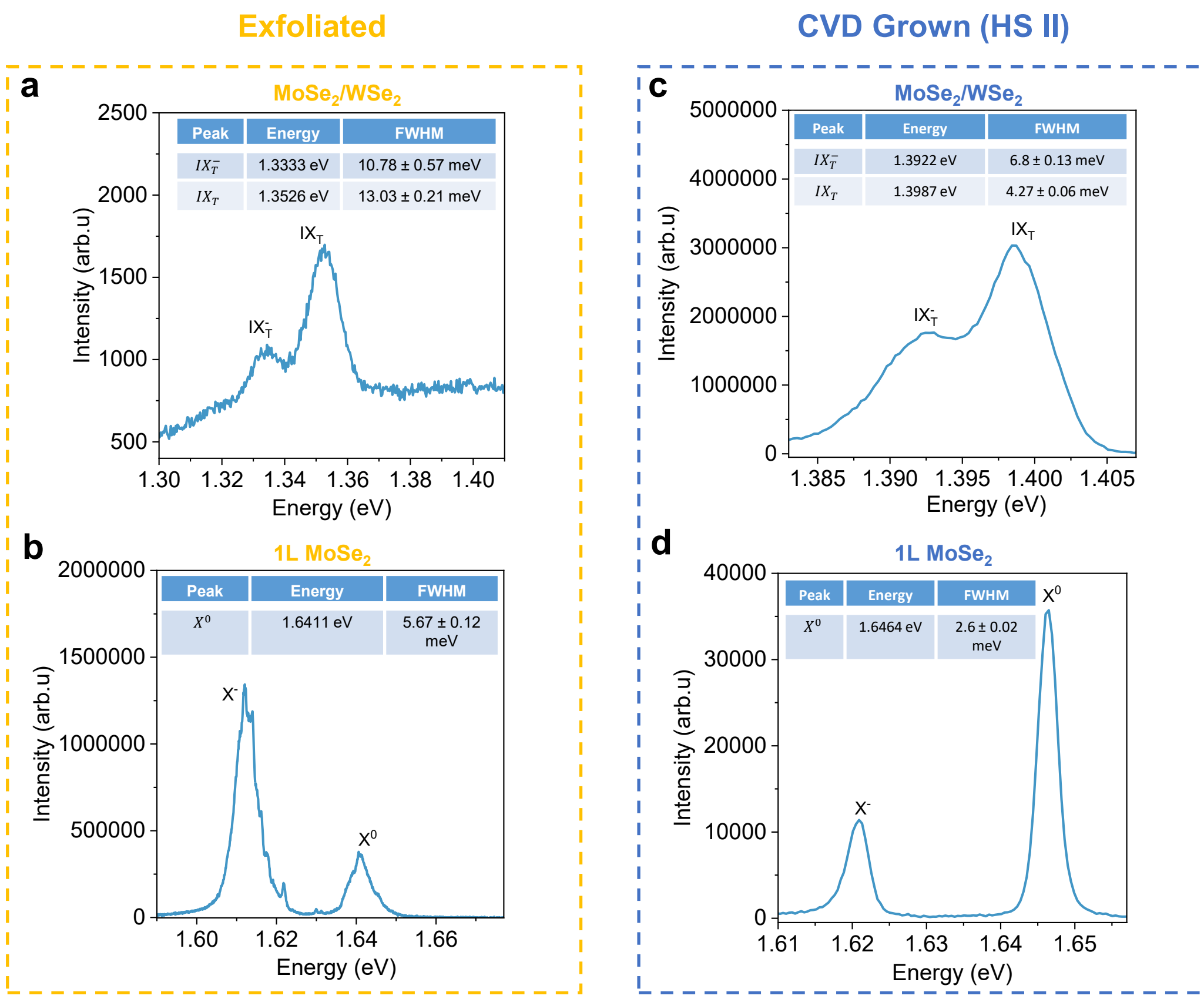

Figure S22: Comparison of PL spectra of the exfoliated sample and the CVD-grown $MoSe_2$-WSe_2 HS II at 4K.

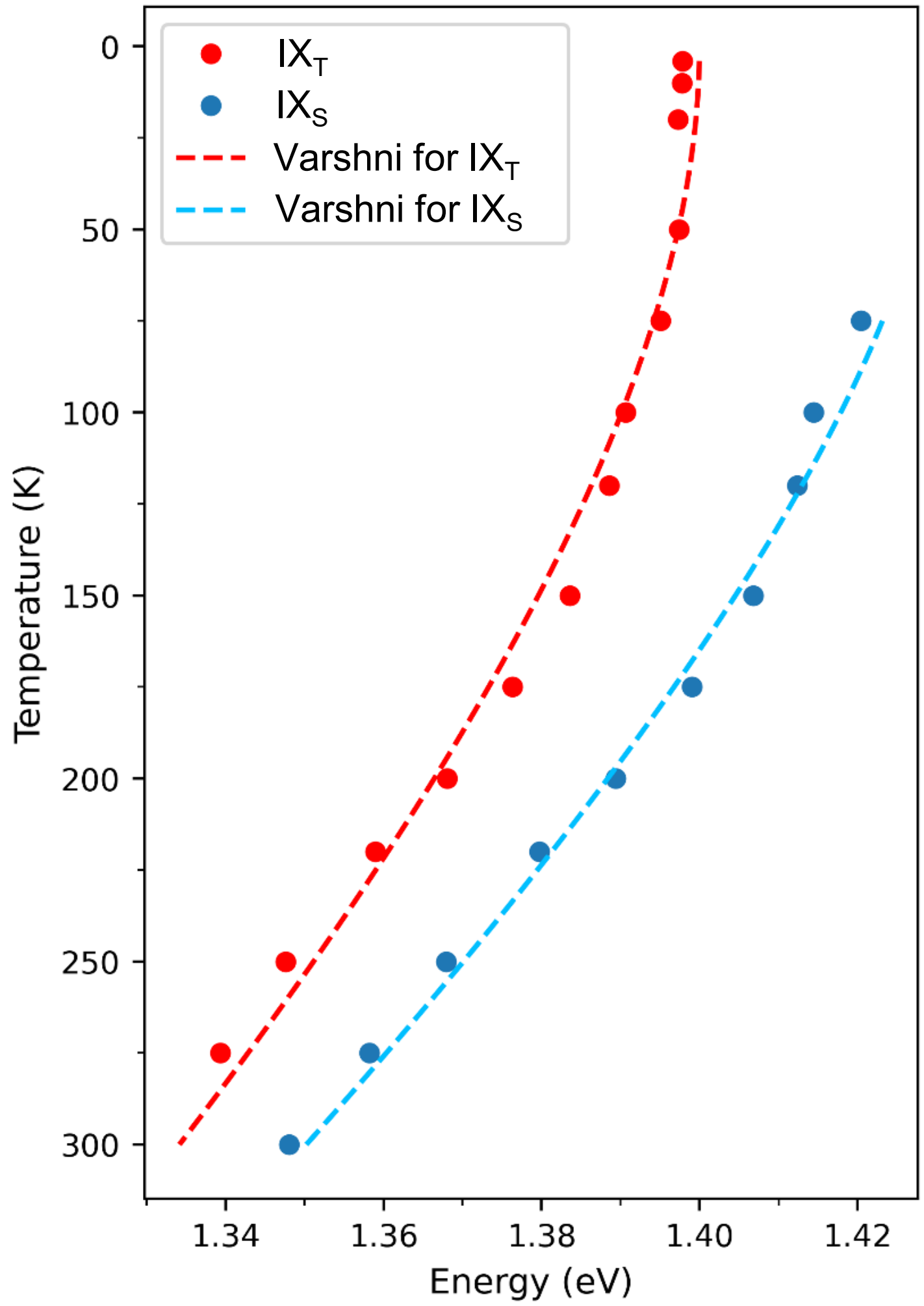

Figure S23: Varshni function fits for the temperature dependent peak shifts of IX_T (in red) and IX_s (in blue).

Table S1: Ratio and molar concentrations of transition metal precursors in aqueous solution.

Ratio C1/C2	Na_2MoO_4 [mM]	Na_2WO_4 [mM]
1:20	2.45	49.0
1:10	2.45	24.5
1:3	2.45	7.35
2:3	4.90	7.35
2:10	4.90	24.5
3:1	7.35	2.45
3:2	7.35	4.90
3:3	7.35	7.35
3:10	7.35	24.5

Table S2: Varshni parameters for 1L-$MoSe_2$, IX_T, IX_s from the HS II sample and from exfoliated 1L-WSe_2 for reference.

Sample	E_0 [eV]	α [eV/K]	β [K]
$MoSe_2$ (HS II)	1.64	9.3E-4	754
WSe_2 (Exfoliated)	1.72	6.0E-4	331
IX_T (HS II)	1.40	5.7E-4	479
IX_s (HS II)	1.43	5.7E-4	335

REFERENCES

[1] H. Kim, G. H. Han, S. J. Yun, J. Zhao, D. H. Keum, H. Y. Jeong, T. H. Ly, Y. Jin, J.-H. Park, B. H. Moon, S.-W. Kim, Y. H. Lee, *Nanotechnol.* **2017**, *28*, 36LT01.

[2] K. Takanabe, S. Shahid, *AlChE J.* **2017**, *63*, 105.

[3] K. Takanabe, A. M. Khan, Y. Tang, L. Nguyen, A. Ziani, B. W. Jacobs, A. M. Elbaz, S. M. Sarathy, F. Tao, *Angew. Chem. Int. Ed.* **2017**, *56*, 10403.

[4] S. Kikuchi, N. Koga, H. Seino, S. Ohno, *J. Nucl. Sci. Technol.* **2016**, *53*, 682.

[5] R. A. Kalt, A. Arcifa, C. Wäckerlin, A. Stemmer, *Nanoscale* **2023**, *15*, 18871.
